# Pediatric T cell and B cell responses to SARS-CoV-2 infection

**DOI:** 10.1172/jci.insight.196032

**Published:** 2025-09-04

**Authors:** L. Benjamin Hills, Numana Bhat, Jillian H. Hurst, Amber Myers, Thomas W. Burke, Micah T. McClain, Elizabeth Petzold, Alexandre T. Rotta, Nicholas A. Turner, Alba Grifoni, Daniela Weiskopf, Yvonne Dogariu, Genevieve G. Fouda, Sallie R. Permar, Alessandro Sette, Christopher W. Woods, Matthew S. Kelly, Shane Crotty

**Affiliations:** 1 Center for Vaccine Innovation, La Jolla Institute for Immunology, La Jolla, California, USA.; 2 Division of Hematology and Oncology, Department of Pediatrics, UCSD, La Jolla, California, USA.; 3 Department of Pediatrics and; 4 Department of Medicine, Duke University School of Medicine, Durham, North Carolina, USA.; 5 Center for Infectious Disease Diagnostics and Innovation, Duke University, Durham, North Carolina, USA.; 6 Durham VA Healthcare System, Durham, North Carolina, USA.; 7 Division of Infectious Diseases and Global Public Health, Department of Medicine, UCSD, La Jolla, California, USA.; 8 Department of Pediatrics, Weill Cornell Medicine, New York, New York, USA.; 9 Department of Pediatrics, University of Arkansas for Medical Sciences, Little Rock, Arkansas, USA.

**Keywords:** Clinical Research, Immunology, Infectious disease, B cells, COVID-19, T cells

## Abstract

**BACKGROUND:**

Understanding age-associated differences in acute and memory adaptive immunity to SARS-CoV-2 and how they contribute to more favorable outcomes in children is critically important.

**METHODS:**

We evaluated SARS-CoV-2–specific T cell, B cell, and antibody responses in 329 peripheral blood samples collected from nonhospitalized children, adolescents, and adults at 3 time points, including acute and memory time points.

**RESULTS:**

Most children produced robust CD4^+^ T cell responses during infection and developed memory CD4^+^ T cells; however, young children less than 4 years old often had undetectable CD4^+^ T cell responses compared with older children and adults. Young children also generated reduced frequencies of memory B cells; despite this, they mounted substantial and durable neutralizing antibody responses. CD4^+^ T cell responses in children were biased toward non-spike epitopes, especially in asymptomatic cases. Memory B cells in children were preferentially classical memory or, paradoxically, CXCR3^+^.

**CONCLUSION:**

These findings support the concept that the kinetics and composition of T and B cell responses shift across age groups and may be associated with milder COVID-19 outcomes in children.

**FUNDING:**

NIH National Institute of Allergy and Infectious Diseases (NIAID) award AI142742, the Duke University School of Medicine, and grants from the Children’s Miracle Network Hospitals, the Translating Duke Health Children’s Health and Discovery Initiative, the NIH NIAID (R01-AI161008-02), and the Defense Advanced Research Projects Agency N66001-09-C-2082. NIH Career Development Awards (K23-AI135090 and K01-AI173398). NIH contract 75N93019C00065.

## Abstract 2

Most children develop robust, multifunctional CD4^+^ T cell, memory B cell, and humoral responses to SARS-CoV-2 infection whereas children &lt;4 years old have uniquely reduced CD4^+^ T cell and memory B cell responses yet maintain humoral responses comparable to those in older individuals.

## Graphical abstract

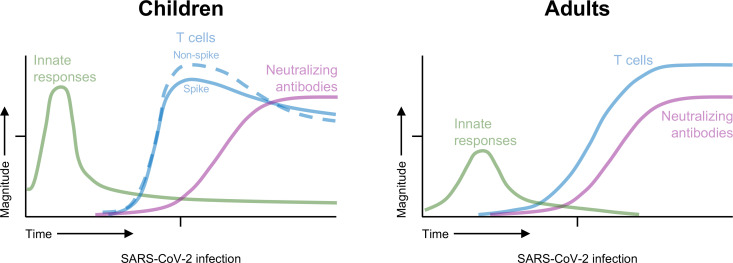

## Introduction

Children infected by the original strain of SARS-CoV-2 typically develop asymptomatic or mildly symptomatic disease (1, 2). Understanding why severe and fatal COVID-19 infections are much less common among children than adults is of high interest. The reduced susceptibility of children to severe COVID-19 is likely determined by a combination of immunological and nonimmunological differences; however, the exact nature of those differences is not fully understood. Children have higher baseline levels of viral sensing innate immune pathways in their upper respiratory tract than adults (3, 4), likely resulting in stronger local antiviral immunity during early infection. In contrast, initial innate immune responses against SARS-CoV-2 in adults are typically suboptimal and further decrease with advancing age (4, 5). While effective innate immune responses contribute to protection, multiple layers of immunity, including adaptive immune responses, are likely involved in controlling SARS-CoV-2 infections (6, 7).

Antibodies clearly provide protection against SARS-CoV-2 (8, 9), but neutralizing antibody responses to SARS-CoV-2 infection are delayed compared with other immune responses. T cell responses in SARS-CoV-2–infected adults are associated with protection from severe COVID-19 (10–17). Children with mild or asymptomatic infection produce a neutralizing antibody response lasting several months, indicating a T cell component in their protective immunity (18, 19). Several studies of SARS-CoV-2–specific T cell responses in children found lower frequencies of SARS-CoV-2–specific memory T cells compared with adults (20–23). Prior studies have primarily evaluated pediatric T cell responses after the acute phase of infection; however, studies conducted in SARS-CoV-2–infected adults demonstrate that rapid, early T cell responses correlate with less severe disease (13). Thus, studying SARS-CoV-2–elicited T cells early after infection is critical for understanding protective immunity in children.

How memory B cells (B_Mem_) shape the antibody response to SARS-CoV-2 infection in children — and how these responses are distinct from those in adults — is also important for understanding pediatric immunity to SARS-CoV-2 (24). One plausible hypothesis is that the heightened innate immunity in children restricts viral replication, lowering viral loads and reducing the magnitude of T cell responses, with antibodies primarily generated via extrafollicular, T-independent B cell responses. Alternatively, children may mount a rapid T cell response to acute SARS-CoV-2 infection because of faster priming from professional antigen-presenting cells activated during rapid pediatric innate immune responses, thereby promoting T-dependent B cell responses that generate high-affinity neutralizing antibodies. These concepts likely apply more broadly to viral infections beyond SARS-CoV-2. Therefore, defining acute T cell and B_Mem_ responses is crucial to clearly understand why children have more favorable outcomes following SARS-CoV-2 infection.

## Results

### SARS-CoV-2–infected pediatric and adult cohort.

We evaluated SARS-CoV-2–specific adaptive immune responses in 329 peripheral blood mononuclear cell (PBMC) samples collected from 183 nonhospitalized children, adolescents, and adults. T cell responses were assessed in 267 PBMC samples across 3 time points, and B_Mem_ responses were evaluated in an additional 62 PBMC samples. A notable feature of this study design was the collection of PBMC samples from outpatient children and adults with acute SARS-CoV-2 infection. Samples for T cell analysis were collected a median of 7 days (acute infection), 59 days (2 months, convalescence time point), and 186 days (6 months, immune memory time point) (Supplemental Figure 1, B and C; supplemental material available online with this article; https://doi.org/10.1172/jci.insight.196032DS1) following PCR-based diagnosis of SARS-CoV-2 infection. Samples for B_Mem_ analysis were collected a median of 178 days following PCR-based diagnosis of SARS-CoV-2 infection. Pediatric samples from children and adolescents who had enrolled in the Biospecimens from Respiratory Virus-Exposed Kids (BRAVE Kids) study between April 2020 and June 2021 were grouped into age ranges 0–3, 4–11, 12–15, and 16–20 years. The BRAVE Kids participants were asymptomatic (15%) or had mild symptoms (85%), such as fever, cough, and headache (25). Adults (≥21 years, median age of 42.4 years for T cell analyses and median age of 45.5 years for B_Mem_ analyses) had enrolled in the Duke University Molecular and Epidemiological Study of Suspected Infection (MESSI) study during the same time period and reported mild symptoms (26). None of the infected pediatric or adult participants were immunized with a SARS-CoV-2 vaccine, were treated with antiviral therapy, or required hospitalization upon infection. Samples from 19 uninfected children and 8 uninfected (SARS-CoV-2 PCR negative) adults were included to provide a baseline for immune response comparisons. Demographic and sample collection data are shown in Supplemental Tables 1 and 2.

### Magnitude and kinetics of SARS-CoV-2–specific CD4^+^ T cells in children.

**Figure 1:**
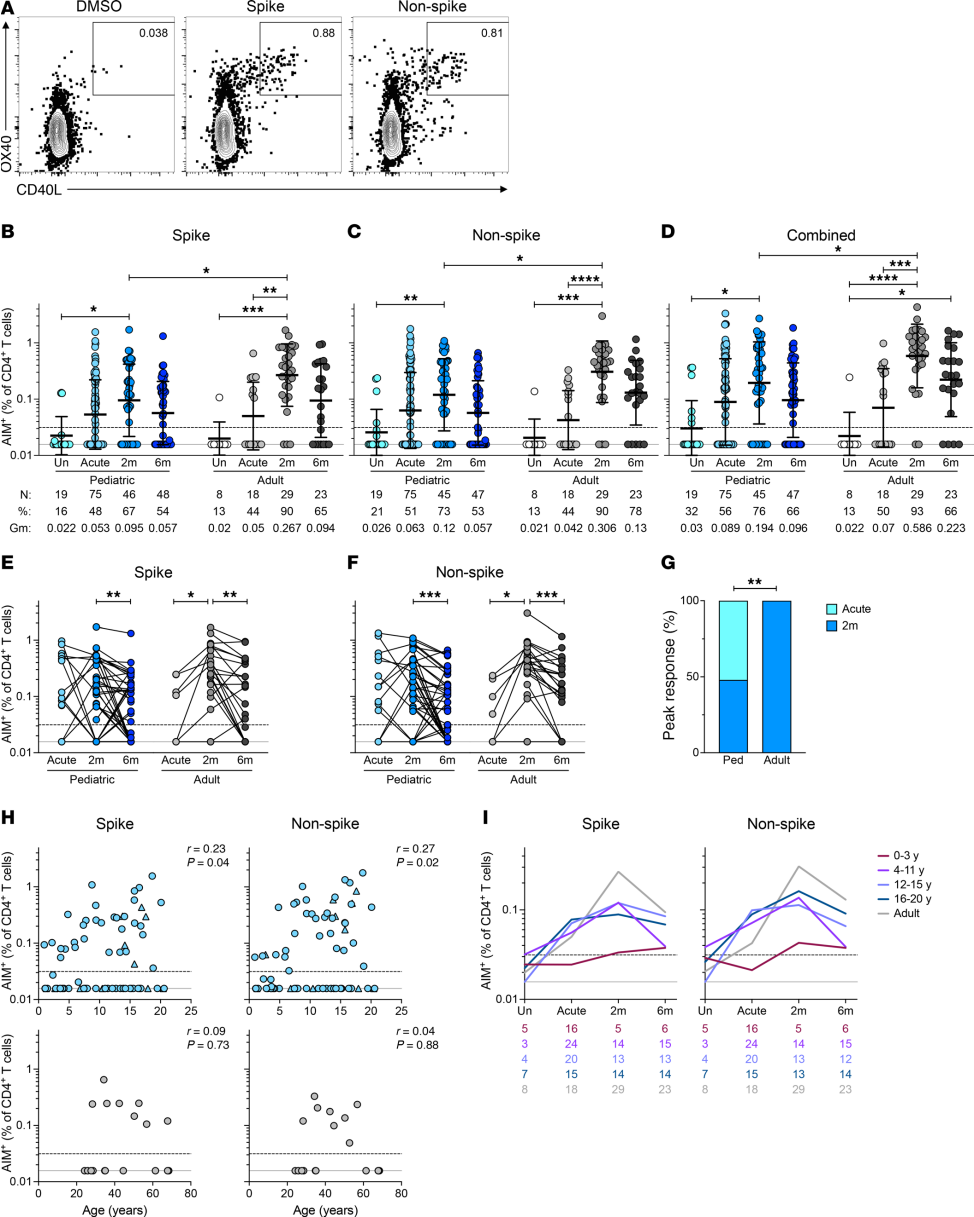
Virus-specific CD4^+^ T cell responses to SARS-CoV-2 infection in children and adults. (**A**) Representative flow cytometry plots showing SARS-CoV-2–specific CD4^+^ T cells assessed by AIM assay. (**B**) Frequencies of SARS-CoV-2 spike-specific, (**C**) non-spike-specific, and (**D**) combined spike and non-spike CD4^+^ T cells. (**E**) Paired longitudinal spike-specific and (**F**) non-spike-specific CD4^+^ T cell responses. (**G**) Bar graph indicating peak responses at acute or 2-month time points, tabulating spike- and non-spike-specific CD4^+^ T cell responses from 14 children and 6 adults (*n* = 25, *n* = 12 responses, respectively). (**H**) Correlations of spike (left) and non-spike (right) CD4^+^ T cell frequencies during acute infection with age in children (top) and adults (bottom). Triangles represent asymptomatic donors. (**I**) Frequencies of SARS-CoV-2 spike- (left) and non-spike-specific (right) CD4^+^ T cells. Connecting lines represent the geometric mean (GeoMean); numbers below the graphs denote the number of donors for each time point. Center lines and error bars in **B**–**D** represent the GeoMean ± geometric SD. Dotted line in **B**–**F**, **H**, and **I** indicates the LOQ; solid gray line indicates the lower limit of detection (LOD). Uninfected participants indicated as Un; 2-month and 6-month time points as 2m and 6m. AIM^+^ denotes the frequencies of OX40^+^CD40L^+^ cells among total CD4^+^ T cells. *N*, %, and Gm in **B**–**D** represent number of donors, percent responders, and GeoMean for each group, respectively. Ped in **G** represents the pediatric group. *P* values for **B**–**D** were calculated by Kruskal-Wallis test with Dunn’s correction for comparisons within the pediatric and adult groups and by Mann-Whitney test with Holm-Šídák correction for comparisons across age groups, for **E** and **F** by Wilcoxon’s test, and for **G** by Fisher’s exact test and are indicated as *P < 0.05, **P < 0.01, ***P < 0.001, ****P < 0.0001. *r* values in **H** indicate Spearman correlation coefficient.

To assess the magnitude and kinetics of SARS-CoV-2–specific CD4^+^ T cell responses in infected children and adults, we measured the frequencies of CD4^+^ T cells by activation-induced marker assay (AIM) (27–31) upon stimulation with SARS-CoV-2 peptide pools spanning the spike protein (spike) (32) or consisting of experimentally defined epitopes from the remainder of the SARS-CoV-2 proteome (non-spike) (33) (OX40^+^CD40L^+^; Figure 1A and Supplemental Figure 1).

There was comparable induction of spike-specific CD4^+^ T cells in children and adults, with 48% and 44% of individuals producing responses above background, respectively (Figure 1B). Collection times at the acute phase of infection (days after symptom onset) were comparable between the groups (Supplemental Figure 1, B and C). At 2 months postinfection, spike-specific CD4^+^ T cells significantly increased from the acute phase in adults but not in children (children: 1.8-fold, *P* = 0.199; adults: 5.3-fold, *P* = 0.0018), with adult responses significantly higher than those in children (*P* = 0.01, Figure 1B). Spike-specific memory CD4^+^ T cells were maintained for at least 6 months after infection in the majority of children (54%) and adults (65%; Figure 1B).

Non-spike–specific CD4^+^ T cells during acute infection were also comparable between children and adults, with 51% and 44% of individuals producing responses above background, respectively (Figure 1C). Non-spike–specific CD4^+^ T cells significantly increased from the acute phase in adults but not in children (children: 1.9-fold, *P* = 0.15; adults: 7.2-fold, *P* < 0.0001), with responses in adults significantly higher than those in children (*P* = 0.025, Figure 1B). Similar to the spike-specific T cells, non-spike–specific memory CD4^+^ T cells were detectable at 6 months postinfection in the majority of children (53%) and adults (78%) (Figure 1C).

Total SARS-CoV-2–specific CD4^+^ T cell responses were calculated by combining the spike and non-spike CD4^+^ T cell responses (Combined, Figure 1D). When examining combined T cell responses, 56% of children had detectable acute CD4^+^ T cell responses, 76% had memory T cells 2 months postinfection, and 66% had memory CD4^+^ T cells 6 months postinfection (Figure 1D). Memory T cell frequencies at 2 months postinfection were lower in children than adults (*P* = 0.017. Figure 1D).

To further assess T cell response kinetics, analysis was performed on the subset of participants who provided longitudinal samples (44 children: Acute = 14, 2m = 44 [non-spike 42], 6m = 44 [non-spike 43]; and 24 adults: Acute = 7, 2m = 24, 6m = 22). Paired longitudinal analysis indicated that SARS-CoV-2–specific CD4^+^ T cell responses in children and adults followed different kinetics (Figure 1, E and F). T cell frequencies in children did not significantly increase between the acute and 2-month time points. In contrast, T cell frequencies in adults were significantly higher at the 2-month time point than the acute time point (children: *P* = 0.6 spike, *P* = 0.3 non-spike; adults: *P* = 0.03 for both spike and non-spike responses; Figure 1, E and F). These trends remained the same when only including participants with samples at all 3 time points (Supplemental Figure 2, A–C). Considering spike or non-spike CD4^+^ T cell responses in aggregate for donors who provided samples at both the acute and 2-month time points, responses peaked at the acute time point more often in children than adults (52%, 13 of 25, versus 0%, 0 of 12, for children and adults, respectively; Fisher’s exact *P* = 0.002; Figure 1G).

For children, the magnitude of T cell responses at the acute time point positively correlated with age (Figure 1H), with non-spike responses showing a marginally stronger correlation (*r* = 0.27, *P* = 0.02) than spike responses (*r* = 0.23, *P* = 0.04), consistent with a previous study (20). Interestingly, SARS-CoV-2–specific CD4^+^ T cell responses in children less than 4 years of age were distinct from all other age groups, remaining below or near the lower limit of quantification (LOQ) at all time points (Figure 1I and Supplemental Figure 2, E–G). Collection times (days after symptom onset) were comparable between this group and older children (Supplemental Figure 1C). Conclusions were similar when analyses comparing T cell frequencies between children and adults were repeated after excluding data from children 0–3 years old (Supplemental Figure 2, H–J).

These experiments showed that SARS-CoV-2–specific CD4^+^ T cell responses were detectable in most infected children and adolescents. In children, circulating CD4^+^ T cell responses most often peaked during acute infection, and the magnitude of the CD4^+^ T cell responses increased with age. Notably, most children less than 4 years of age had undetectable circulating SARS-CoV-2–specific CD4^+^ T cells at all time points.

### SARS-CoV-2 infection in children induces a stronger acute non-spike CD4^+^ T cell response.

**Figure 2:**
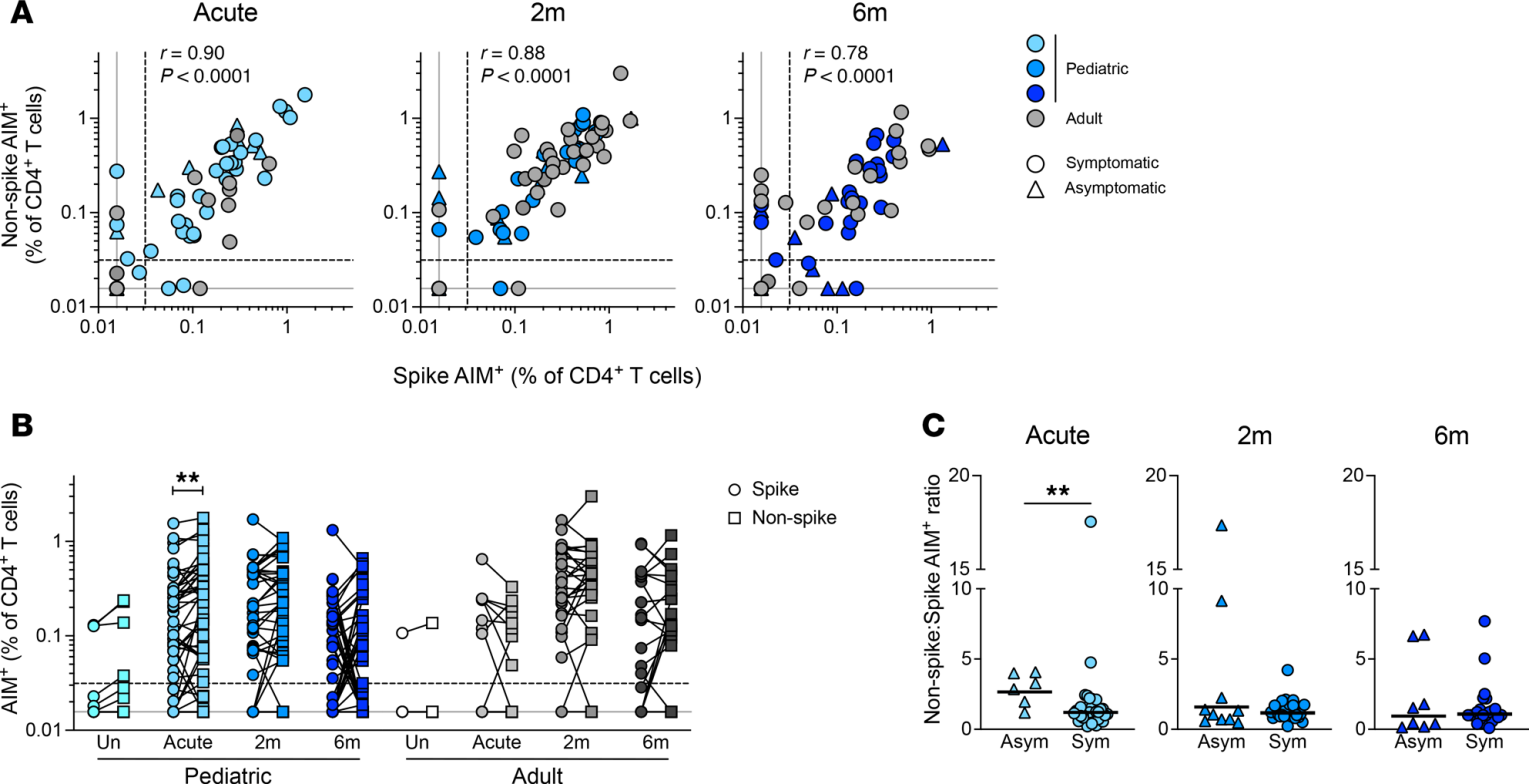
SARS-CoV-2 infection in children induces a bias toward acute non-spike CD4^+^ T cell responses. (**A**) Correlation of spike-specific CD4^+^ T cell responses with non-spike-specific CD4^+^ T cell responses. Triangles denote asymptomatic infections. (**B**) Paired spike- and non-spike-specific CD4^+^ T cell responses at indicated time points; children (*n*): Un = 19, Acute = 75, 2m = 45, 6m = 46; adults (*n*): Un = 8, Acute = 18, 2m = 29, 6m = 23. (**C**) Comparisons of ratios of non-spike/spike-specific CD4^+^ T cell responses between asymptomatic (Asym) and symptomatic (Sym) pediatric donors at indicated time points; Acute: Asym = 6, Sym = 36; 2m: Asym = 10, Sym = 24; 6m: Asym = 8, Sym = 23. Center lines in **C** represent the GeoMean. Dotted line in **A** and **B** indicates the LOQ of the assay; solid gray line indicates the lower LOD. Uninfected participants are indicated as Un; 2-month and 6-month time points as 2m and 6m, respectively. AIM^+^ denotes the frequencies of OX40^+^CD40L^+^ among total CD4^+^ T cells. *r* values in **A** indicate Spearman correlation coefficient. *P* values for **B** were calculated by Wilcoxon’s test with Holm-Šídák correction and for **C** by Mann-Whitney test and are indicated as **P < 0.01.

A faster and stronger innate immune response to SARS-CoV-2 in children compared with adults could cause differences in SARS-CoV-2 antigen presentation to CD4^+^ T cells; in turn, a rapid innate immune response could bias CD4^+^ T cell responses toward distinct SARS-CoV-2 proteins or epitopes. We therefore compared the magnitudes of spike- and non-spike–specific CD4^+^ T cell responses. While we observed a strong correlation between spike and non-spike responses at all time points in both children and adults (each *P* < 0.0001), the responses in children appeared to skew toward non-spike CD4^+^ T cell responses during acute infection (Figure 2A). This was verified by direct comparison of paired non-spike- and spike-specific CD4^+^ T cell frequencies within individuals (*P* = 0.002, Figure 2B).

Children analyzed in this study were mildly symptomatic or asymptomatic. An association was previously reported between a higher non-spike to spike T cell response specificity ratio and less severe COVID-19 in a small adult cohort (15). To determine if higher non-spike-specific CD4^+^ T cell responses in children were associated with less severe disease, we compared spike and non-spike CD4^+^ T cell frequencies among asymptomatic and symptomatic children. The non-spike/spike T cell ratio was significantly higher among asymptomatic participants compared with symptomatic participants during acute infection (2.2-fold, *P* = 0.006, Figure 2C). Overall, these results suggest that CD4^+^ T cell responses preferentially targeting non-spike proteins are associated with asymptomatic SARS-CoV-2 infection.

### CD4^+^ T cell functionalities in children.

**Figure 3:**
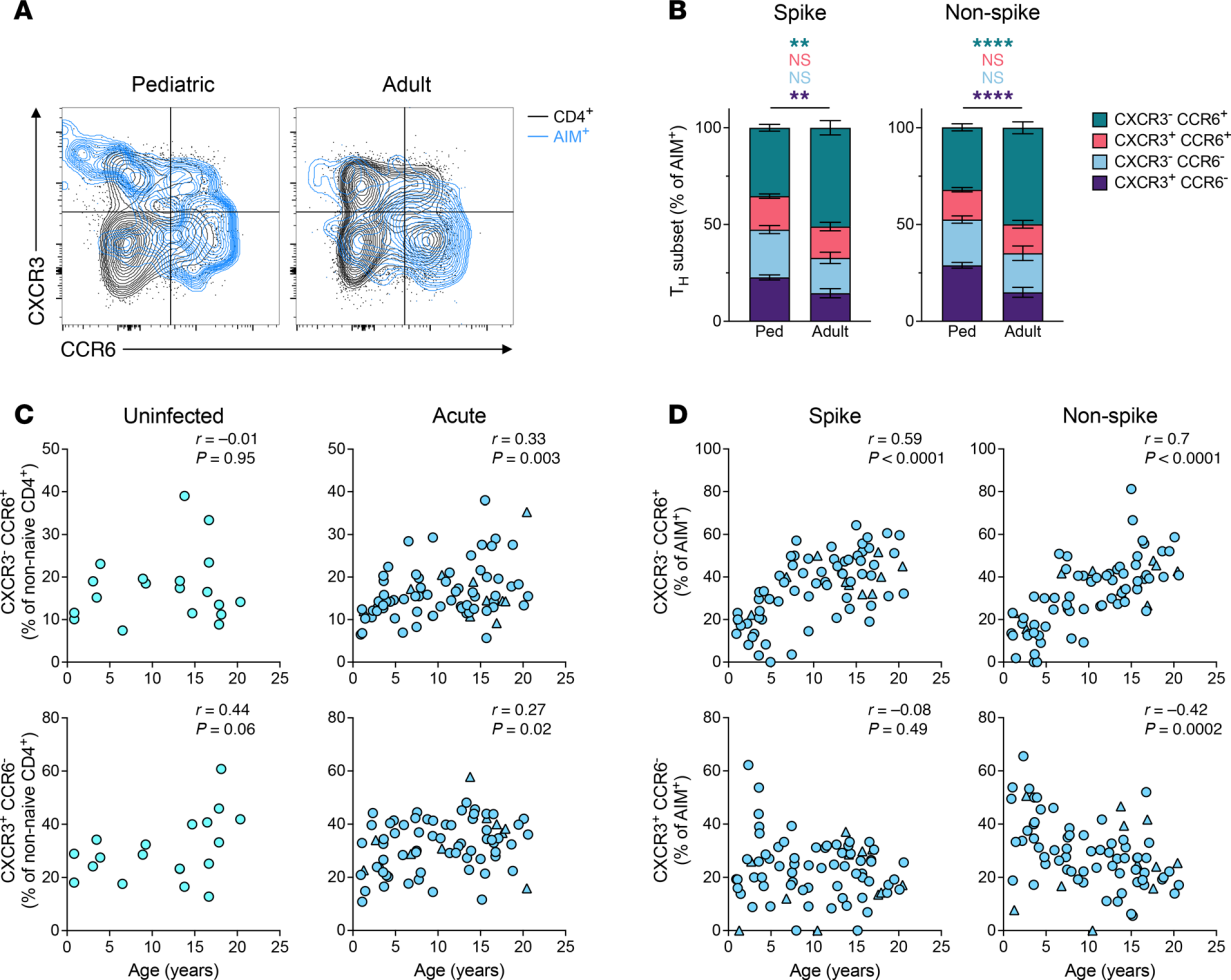
T helper subset distribution in SARS-CoV-2–infected children and adults. (**A**) Representative flow cytometry plots showing T helper (T_H_) subset distribution among total CD4^+^ T cells (black) and among SARS-CoV-2 antigen-specific CD4^+^ T cells (blue) in children (left) and adults (right). (**B**) Proportion plots showing distribution of T_H_ subsets among SARS-CoV-2 spike- (left) and non-spike-specific (right) CD4^+^ T cells at the acute phase of infection. (**C** and **D**) Correlations of CCR6^+^ (top) and CXCR3^+^ frequencies (bottom) with age; *y*-axis represents percentages of (**C**) total non-naive CD4^+^ T cells and (**D**) SARS-CoV-2–specific CD4^+^ T cells. Columns and error bars in **B** represent the mean and SEM, respectively. Ped in **B** represents the pediatric group. *P* values for **B** were calculated by Mann-Whitney test with Holm-Šídák correction and are indicated as **P < 0.01, ****P < 0.0001. *r* in **C** and **D** indicates Spearman correlation coefficient.

We next evaluated the expression of chemokine receptors associated with additional T helper cell subsets using differential staining of chemokine receptors CXCR3 and CCR6 in total CD4^+^ T cells (Supplemental Figure 3) and SARS-CoV-2–specific CD4^+^ T cells (Figure 3, A and B). CXCR3^–^CCR6^+^ CD4^+^ T cells (CCR6^+^) formed the largest proportion of AIM^+^CD4^+^ T cells in both children and adults (Figure 3B); however, AIM^+^CCR6^+^ cells were reduced in children compared with adults during the acute phase of infection (spike-specific: *P* = 0.001; non-spike-specific: *P* < 0.0001; Figure 3B). We observed a positive correlation between the frequency of CCR6^+^CD4^+^ T cells and age among children, including total (*r* = 0.33, *P* = 0.003) and SARS-CoV-2–specific cells (spike: *r* = 0.59, *P* < 0.0001; non-spike: *r* = 0.7, *P* < 0.0001) (Figure 3, C and D). Compared with adults, children had higher frequencies of spike- and non-spike–specific CXCR3^+^CCR6^–^CD4^+^ T cells (CXCR3^+^) during the acute phase of infection (spike: *P* = 0.008, non-spike: *P* < 0.0001; Figure 3B). In contrast with CCR6^+^CD4^+^ T cells, we observed that SARS-CoV-2–specific CXCR3^+^CD4^+^ T cell frequencies decreased with age within the pediatric group (non-spike: *r* = –0.42, *P* = 0.0002; Figure 3D) despite an increase in total CXCR3^+^CD4^+^ T cells with age (*r* = 0.27, *P* = 0.02; Figure 3C).

**Figure 4:**
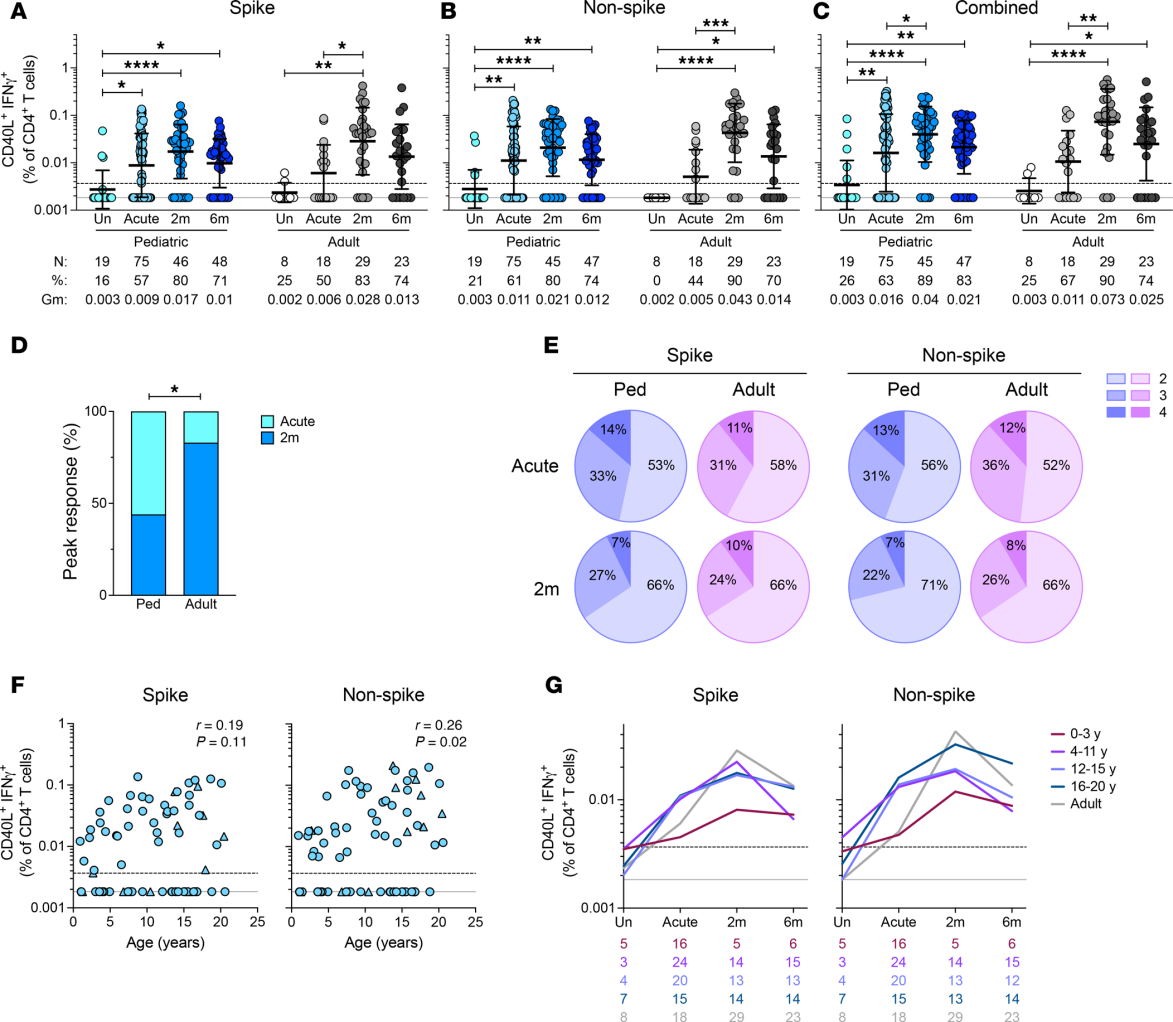
SARS-CoV-2–specific CD4^+^ T cell cytokine responses in children. (**A**) Frequencies of SARS-CoV-2 spike-specific, (**B**) non-spike-specific, and (**C**) spike and non-spike combined IFN-γ–producing CD4^+^ T cells. (**D**) Bar graph indicating peak responses at acute or 2-month time points, tabulating spike- and non-spike-specific IFN-γ^+^CD4^+^ T cell responses from 14 children and 7 adults (*n* = 27, *n* = 12 responses, respectively). (**E**) Pie charts showing the proportion of spike-specific cytokine-producing CD4^+^ T cells bearing 2, 3, or 4 functions. (**F**) Correlations of spike (left) and non-spike (right) CD40L^+^IFN-γ^+^CD4^+^ T cell frequencies at the acute phase of infection with age. Triangles represent asymptomatic donors. (**G**) Frequencies of SARS-CoV-2 spike-specific (left) and non-spike specific (right) CD40L^+^IFN-γ^+^CD4^+^ T cells. Each connecting line represents the GeoMean; numbers below the graphs denote the number of donors for each time point. Center lines and error bars in **A**–**C** represent the GeoMean ± geometric SD. Dotted line in **A**–**C**, **F**, and **G** indicates the LOQ; solid gray line indicates the lower LOD. Uninfected participants are indicated as Un; 2-month and 6-month time points as 2m and 6m. *N*, %, and Gm in **A**–**C** represent number of donors, percent responders, and GeoMean for each group, respectively. Ped in **D** represents the pediatric group. *P* values for **A**–**C** were calculated by Kruskal-Wallis test with Dunn’s correction for comparisons within the pediatric and adult groups and by Mann-Whitney test with Holm-Šídák correction for comparisons across pediatric and adult age groups and for **D** by Fisher’s exact test and are indicated as *P < 0.05, **P < 0.01, ***P < 0.001, ****P < 0.0001. *r* in **F** indicates Spearman correlation coefficient.

We further assessed CD4^+^ T cell functionalities by measuring cytokine expression using a hybrid AIM and intracellular cytokine staining (ICS) assay. A significant induction of IFN-γ–producing spike-specific CD4^+^ T cells was observed for children in the acute phase of infection (3-fold, *P* = 0.01; Figure 4A). IFN-γ^+^ spike-specific CD4^+^ T cells increased in children and adults at 2 months postinfection (Figure 4A) and were detectable in most children and adults for at least 6 months postinfection (Figure 4A). Substantial frequencies of non-spike-specific IFN-γ^+^CD4^+^ T cells were also observed in both children and adults (Figure 4, B and C). Similar to AIM responses, IFN-γ^+^ responses peaked in the acute phase more often for pediatric participants (56%, 15 of 27) than adults (17%, 2 of 12) (Fisher’s exact *P* = 0.037; Figure 4D). This further supported our observations from AIM assays that CD4^+^ T cell responses peaked earlier in children than in adults (Figure 1G). Paired longitudinal analysis of SARS-CoV-2–specific IFN-γ^+^CD4^+^ T cell responses showed no significant difference between spike-specific responses at the acute and 2-month time points in children or adults, whereas adult non-spike responses increased at the 2-month time point (Supplemental Figure 4A).

SARS-CoV-2–specific CD4^+^ T cells expressing granzyme B, IL-2, or TNF were induced with similar trends during the acute and convalescent phases in comparison with IFN-γ^+^CD4^+^ T cells (Supplemental Figure 4, C–E). In addition, we assessed multifunctionality of SARS-CoV-2–specific CD4^+^ T cells based on combinations of IFN-γ, TNF, and/or IL-2 coexpressed with CD40L (Figure 4E). Overall, the frequency of polyfunctional SARS-CoV-2–specific CD4^+^ T cells in children was comparable to that observed in adults (Figure 4E).

As with AIM (Figure 1, H and I), we further assessed the correlation of cytokine-producing SARS-CoV-2–specific CD4^+^ T cells with age. Non-spike–specific IFN-γ^+^CD4^+^ T cell frequencies correlated with age, while spike responses were not significantly correlated with age (Figure 4F). Similar to AIM^+^ responses (Figure 1I), IFN-γ^+^ SARS-CoV-2–specific CD4^+^ T cell responses in children under 4 years of age were lower compared with other age groups (Figure 4G).

**Figure 5:**
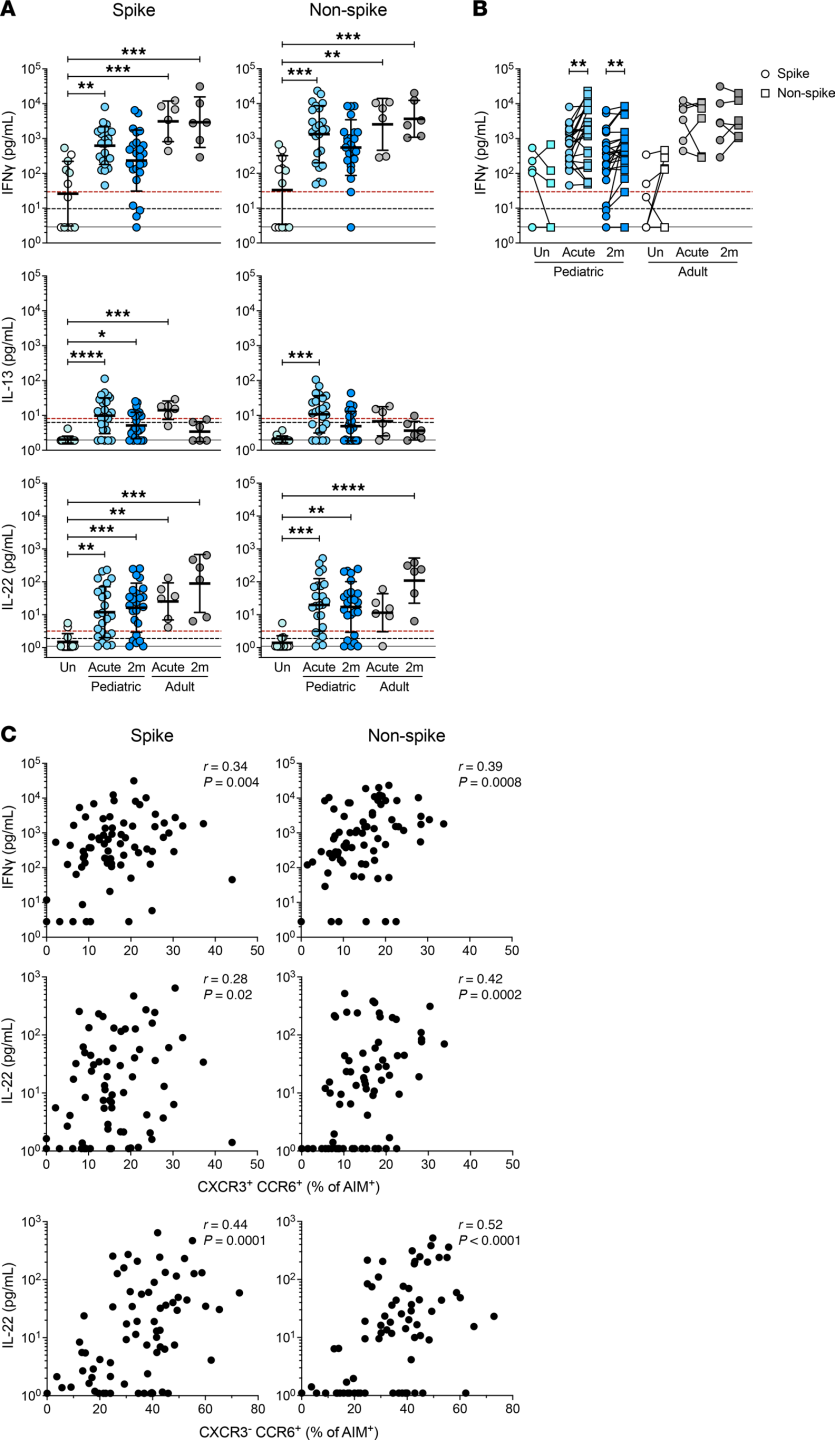
SARS-CoV-2–specific CD4^+^ T cell cytokine secretion in children. (**A**) Spike- and non-spike-specific CD4^+^ T cell secreted cytokine responses. In the uninfected group, blue circles represent pediatric samples, and white circles represent adult samples. (**B**) Paired spike- and non-spike-specific IFN-γ secretion; children *n*: Un = 6, Acute = 24, 2m = 24; adults *n*: Un = 6, Acute = 6, 2m = 6. (**C**) Correlation between secreted cytokine measured by cytokine bead array and the frequency of T_H_ subset determined by hybrid AIM assay. Indicated populations shown as percentage of OX40^+^CD40L^+^CD4^+^ for children and adults in aggregate. Solid black line in **A** and **B** indicates the lower LOD; dashed black line indicates the LOQ; dashed red line indicates the median of all DMSO-negative controls. Uninfected participants are indicated as Un; 2-month and 6-month time points as 2m and 6m. AIM^+^ in **C** denotes the frequencies of OX40^+^CD40L^+^ cells as percentage of CD4^+^ T cells. *P* values for **A** were calculated by Kruskal-Wallis test with Dunn’s correction, and for **B** by Wilcoxon’s test with Holm-Šídák correction and are indicated as *P < 0.05, **P < 0.01, ***P < 0.001, ****P < 0.0001. *r* in **C** indicates Spearman correlation coefficient.

SARS-CoV-2–specific T helper functions were further assessed by quantifying secreted cytokines (Figure 5A and Supplemental Figure 4, B and F–H). Cytokine secretion was strongly correlated with the proportion of AIM^+^CD4^+^ T cells and CD40L^+^IFN-γ^+^CD4^+^ T cells (Supplemental Figure 4B). IFN-γ secretion in children, but not in adults, was higher in response to non-spike peptides than spike peptides, indicating skewing of the antiviral CD4^+^ T cell response toward non-spike epitopes (Figure 5B). Secreted IFN-γ correlated with the frequency of CXCR3^+^CCR6^+^ SARS-CoV-2–specific cells but not CXCR3^+^CCR6^–^ cells (Figure 5C and Supplemental Figure 4F).

We observed IL-22 induction from CD4^+^ T cell responses to both spike and non-spike antigens in children and to spike antigens in adults during the acute phase of infection (Figure 5A). IL-22 levels correlated with the frequency of CXCR3^+^CCR6^+^ and CXCR3^–^CCR6^+^ SARS-CoV-2–specific CD4^+^ T cells (Figure 5C). Secreted IL-13 was detectable in the pediatric group during the acute phase of infection (Figure 5A), and IL-22, but not IL-13, levels correlated with age in the pediatric group (IL-22 *P* = 0.002 and <0.0001; Supplemental Figure 4, G and H).

Overall, children developed a SARS-CoV-2–specific CD4^+^ T cell response early in the course of infection with multiple effector functions, marked by potent production of IFN-γ, TNF, and IL-2, and preferential expression of CXCR3.

### Circulating T follicular helper cell response to SARS-CoV-2 in children.

**Figure 6:**
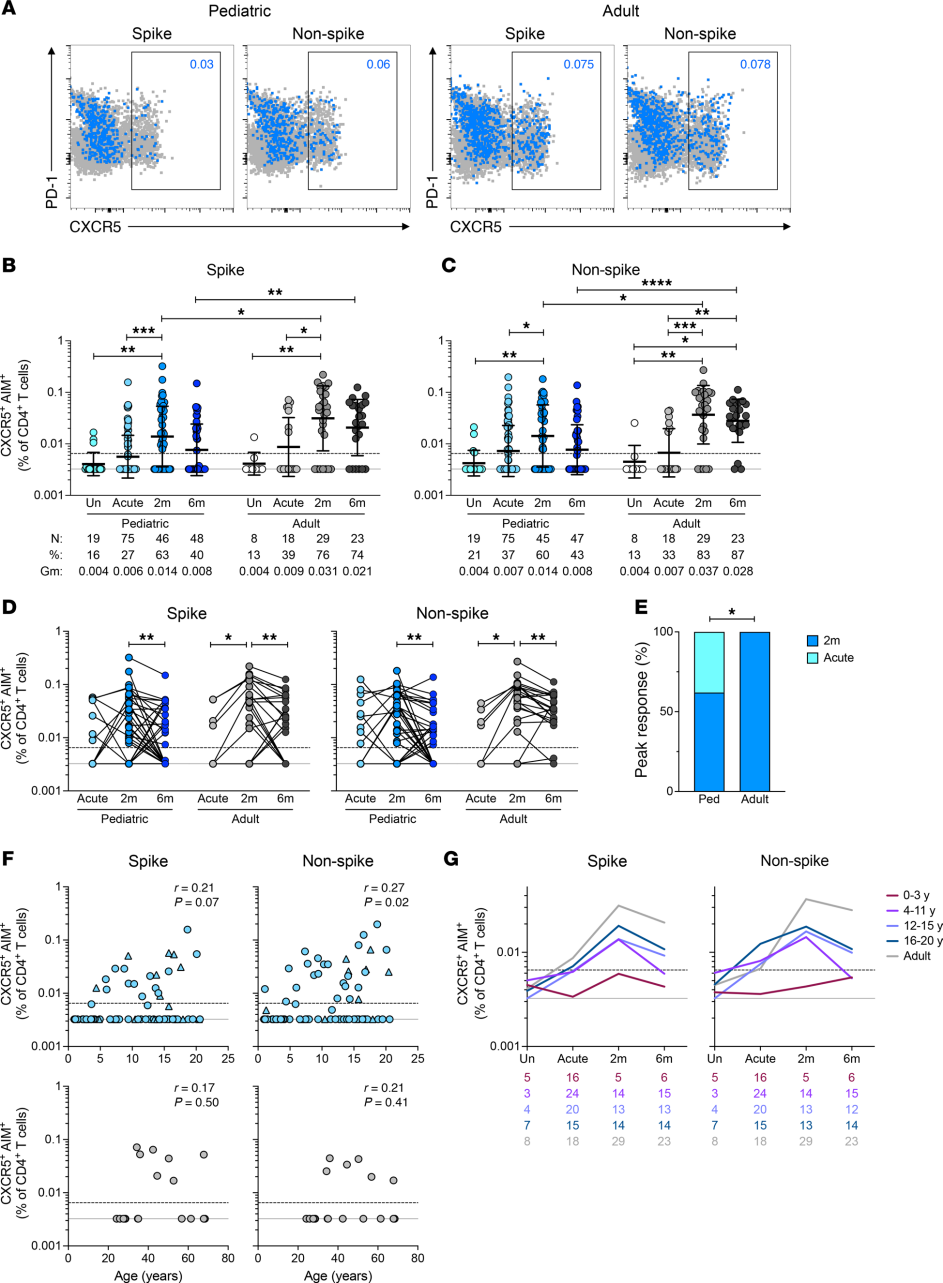
SARS-CoV-2–specific cT_FH_ and antibody response in children. (**A**) Representative flow cytometry plots showing SARS-CoV-2–specific CD4^+^ T cells (blue) overlaid on total CD4^+^ T cells (gray); CXCR5^+^ gate represents cT_FH_ cells. Numbers in blue indicate frequencies of SARS-CoV-2–specific cT_FH_ among total CD4^+^ T cells. PD-1, programmed cell death 1. (**B**) Frequencies of spike-specific and (**C**) non-spike-specific cT_FH_ cells. (**D**) Paired longitudinal spike- (left) and non-spike-specific (right) cT_FH_ responses. (**E**) Bar graph indicating peak responses at acute or 2-month time points, tabulating spike- and non-spike-specific cT_FH_ responses from 12 children and 6 adults (*n* = 21, *n* = 12 responses, respectively). (**F**) Correlations of spike- (left) and non-spike-specific (right) cT_FH_ frequencies at acute phase of infection with age in children (top) and adults (bottom). Triangles represent asymptomatic donors. (**G**) Frequencies of spike-specific (left) and non-spike-specific (right) cT_FH_ cells. Each connecting line represents the GeoMean; numbers below the graphs denote number of donors for each time point. Center lines and error bars in **B** and **C** represent the GeoMean ± geometric SD. Dotted line in **B**–**D**, **F**, and **G** indicates the LOQ; solid gray line indicates the lower LOD. Uninfected participants indicated as Un; 2-month and 6-month time points as 2m and 6m. AIM^+^ denotes frequencies of OX40^+^CD40L^+^ cells among total CD4^+^ T cells. *N*, %, and Gm in **B** and **C** represent number of donors, percent responders, and GeoMean for each group, respectively. Ped in **E** represents the pediatric group. *P* values for **B** and **C** were calculated by Kruskal-Wallis test with Dunn’s correction for comparisons within pediatric and adult groups and by Mann-Whitney test with Holm-Šídák correction for comparisons across age groups, for **D** by Wilcoxon’s test, and for **E** by Fisher’s exact test, indicated as *P < 0.05, **P < 0.01, ***P < 0.001, ****P < 0.0001. *r* in **F** indicates Spearman correlation coefficient.

Circulating T follicular helper (cT_FH_) cells, identified by the expression of the chemokine receptor CXCR5, are critical for providing help to B cells to produce effective antibody responses against viral infections. Spike- and non-spike–specific AIM^+^ (OX40^+^CD40L^+^) SARS-CoV-2–specific cT_FH_ cells were quantified among total cT_FH_ (CXCR5^+^CD4^+^) cells (Figure 6A). Spike-specific cT_FH_ frequencies increased 2.5-fold in children and 3.6-fold in adults at 2 months postinfection (*P* = 0.0004 and = 0.019) and persisted for at least 6 months after infection in both children and adults (Figure 6B). Spike-specific cT_FH_ frequencies were lower in children than in adults at 2 and 6 months postinfection (*P* = 0.038 and = 0.006; Figure 6B). Non-spike-specific cT_FH_ frequencies showed similar patterns (Figure 6C). Paired longitudinal analysis (as in Figure 1, E and F) indicated a significant increase in spike-specific cT_FH_ frequency from the acute to the 2-month time point in adults (*P* = 0.03) but not in children, followed by a decline at the 6-month time point in both adults and children (*P* = 0.005 and 0.008; Figure 6D). SARS-CoV-2–specific cT_FH_ responses peaked in the acute phase of infection more frequently for children than for adults; however, cT_FH_ frequencies peaked at later time points in the majority of both pediatric and adult participants (62% and 100%, Fisher’s exact *P* = 0.03; Figure 6E). This was in contrast with total SARS-CoV-2–specific CD4^+^ T cell responses in children, which more frequently peaked in the acute phase (Figure 1G and Figure 4D). During the acute phase of infection, both spike- and non-spike–specific cT_FH_ frequencies in children increased with age (Figure 6F), as was observed for total SARS-CoV-2–specific CD4^+^ T cells (Figure 1H). Similar to the total SARS-CoV-2–specific CD4^+^ T cell repertoire, SARS-CoV-2–specific cT_FH_ responses in children less than 4 years of age were substantially lower than in all other age groups (Figure 6G and Supplemental Figure 5B).

Total CXCR5^+^CD4^+^ cT_FH_ cell frequencies were similar between adults and children (Supplemental Figure 5C); however, PD-1^+^CXCR5^+^CD4^+^ cT_FH_ frequencies were higher in children at the acute phase compared with adults or uninfected children (Supplemental Figure 5D), indicating that a greater proportion of total cT_FH_ cells are in an activated state in children compared with adults.

In summary, children had lower SARS-CoV-2–specific cT_FH_ frequencies compared with adults, which increased with age within the pediatric cohort. Children less than 4 years of age had distinctly low SARS-CoV-2–specific cT_FH_ cells at all time points.

### B cell memory to SARS-CoV-2 infection in children and adults.

**Figure 7:**
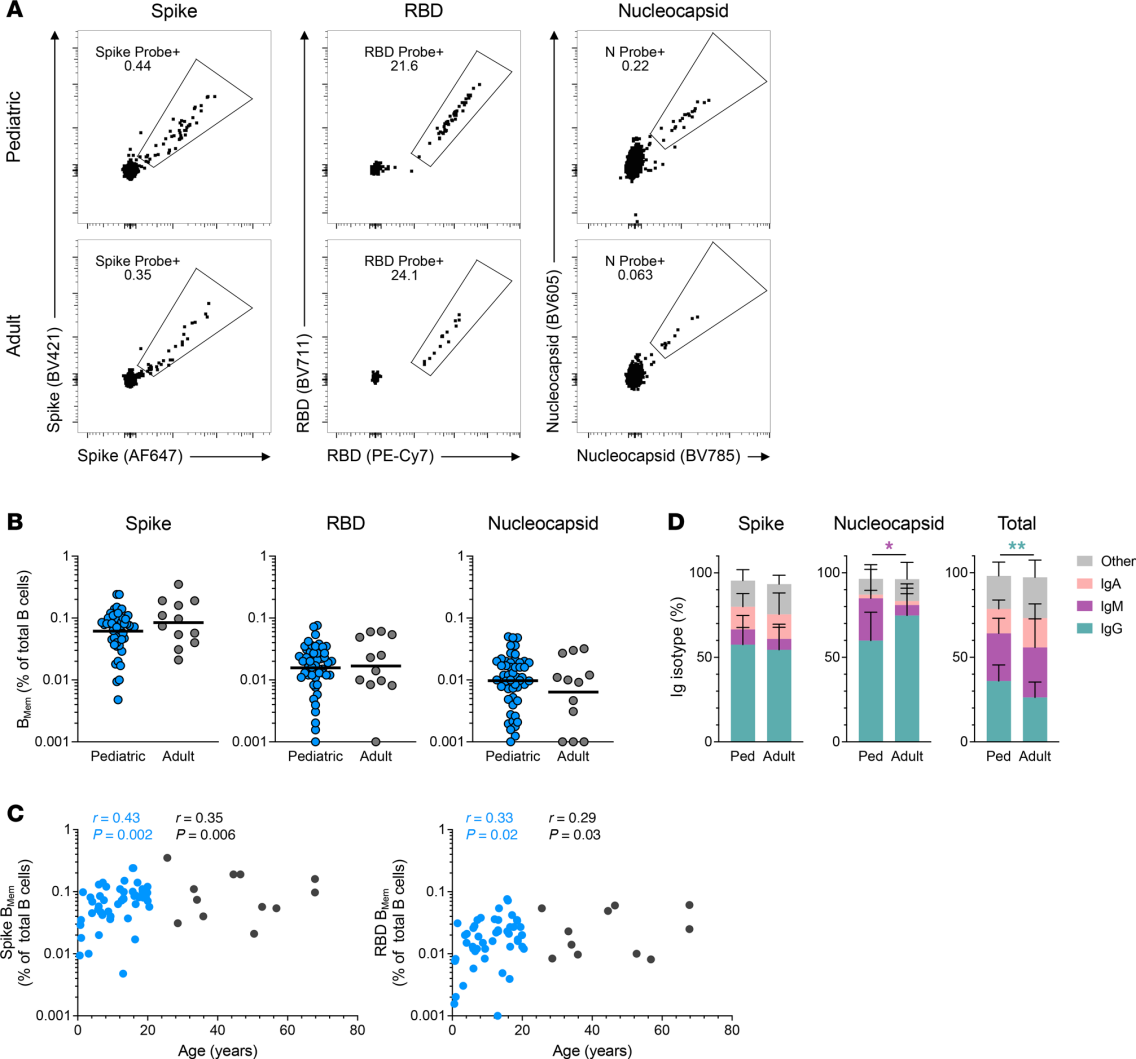
Antigen-specific B cell memory to SARS-CoV-2 infection in children and adults. (**A**) Representative flow cytometry plots showing spike (left), RBD (middle), and nucleocapsid (right) probe binding B_Mem_ in children (*n* = 50) and adults (*n* = 12) at 6 months postinfection. (**B**) Frequencies of spike (left), RBD (middle), and nucleocapsid-binding (right) B_Mem_ shown as percentages of total B cells (CD19^+^CD20^+^). (**C**) Correlations of spike (left) and RBD-binding (right) B_Mem_ frequencies with age, in children (blue) and adults (black). (**D**) Relative frequencies of IgG, IgM, IgA, and other isotypes of B_Mem_ indicated as percentage of spike (left), nucleocapsid (middle), and total (right) B_Mem_. Center lines in **B** represent the GeoMean. Center lines and error bars in **D** represent the mean ± SD. *P* values for **B** were calculated by Mann-Whitney test and for **D** by Mann-Whitney test with Holm-Šídák correction, indicated as *P < 0.05, **P < 0.01. *r* in **C** indicates Spearman correlation coefficient; *r* values in black indicate Spearman correlation coefficient for the combined pediatric and adult data; *r* values in blue indicate Spearman correlation coefficient for the pediatric data alone.

Little is known regarding B_Mem_ responses to SARS-CoV-2 infection in children. We thus quantified SARS-CoV-2–specific B_Mem_ at 6 months postinfection in children and compared them to B_Mem_ in adults. SARS-CoV-2–specific responses were assessed for 3 distinct antigens: spike, spike receptor-binding domain (RBD), and nucleocapsid (Figure 7A). The frequency of spike-, RBD-, or nucleocapsid-specific B_Mem_ among the total B cell pool was equivalent between children and adults (Figure 7B). However, among pediatric donors, there was a correlation between age and frequency of spike- or RBD-specific B_Mem_ (spike: *r* = 0.43, *P* = 0.002; RBD: *r* = 0.33, *P* = 0.02) (Figure 7C). In contrast, there was no correlation for nucleocapsid-specific B cells (Supplemental Figure 6B). There was also no appreciable difference in the isotype frequencies of SARS-CoV-2 spike-specific B_Mem_ between adult and pediatric donors (Figure 7D).

**Figure 8:**
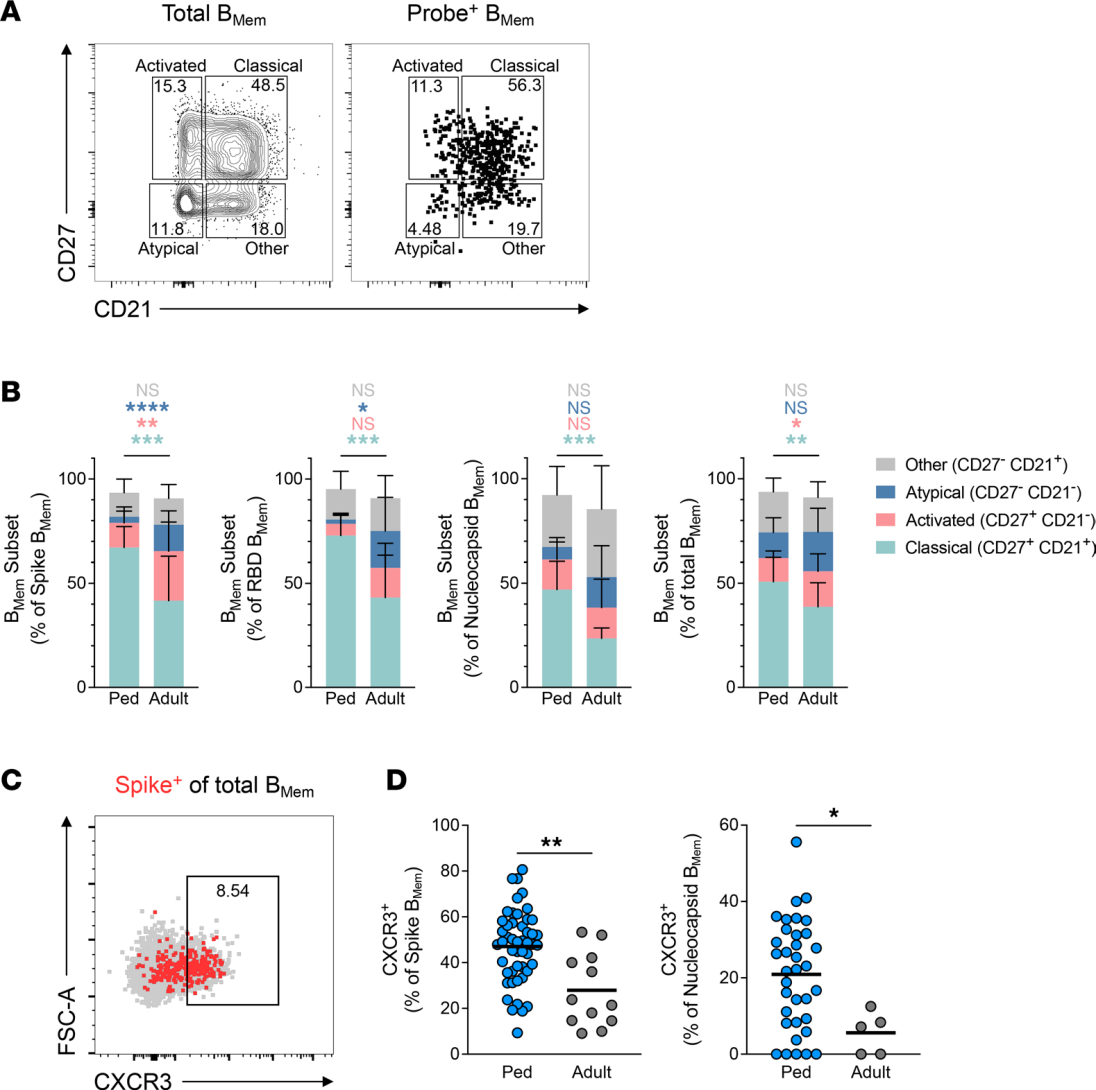
SARS-CoV-2–specific B_Mem_ in children are predominantly classical B_Mem_ and express CXCR3. (**A**) Representative flow cytometry plots showing B_Mem_ subsets based on differential CD27 and CD21 staining, gated on total B_Mem_ (left) and probe-binding B_Mem_ (right). (**B**) Frequencies of B_Mem_ subsets indicated as percentage of SARS-CoV-2–specific or total B_Mem_. (**C**) Representative flow cytometry plot showing CXCR3 staining on SARS-CoV-2–specific B_Mem_. FSC, forward scatter. (**D**) Frequencies of CXCR3^+^ B_Mem_ indicated as percentage of spike- or nucleocapsid-binding B_Mem_; spike: *n* = 49 pediatric group, *n* = 12 adult group; nucleocapsid: *n* = 35 pediatric group, *n* = 5 adult group. Center lines and error bars in **B** represent the mean ± SD. Center lines in **D** represent the mean. Ped in **B** and **D** represents the pediatric group. *P* values for **B** were calculated by Mann-Whitney test with Holm-Šídák correction and for **D** by Mann-Whitney test and are indicated as *P < 0.05, **P < 0.01, ***P < 0.001, ****P < 0.0001.

B_Mem_ comprise phenotypically and functionally distinct subsets, including classical, activated, and atypical B_Mem_, distinguished by differential surface expression of CD27 and CD21 (Figure 8A) (34). Spike-specific B_Mem_ in children were strongly skewed toward a classical (CD27^+^CD21^+^) phenotype, whereas activated (CD27^+^CD21^–^) and atypical (CD27^–^CD21^–^) B_Mem_ were more prevalent in adults (Figure 8B and Supplemental Figure 7A). Similar age-associated differences were observed for RBD- and nucleocapsid-specific B_Mem_ (Figure 8B and Supplemental Figure 7, B and C). Interestingly, CXCR3 was more frequently expressed on pediatric spike-specific B_Mem_ (Figure 8, C and D), which was unexpected, since CXCR3 expression is commonly associated with atypical and activated B_Mem_ (35, 36). However, this did align with our finding that children had higher frequencies of CXCR3^+^ SARS-CoV-2–specific CD4^+^ T cells (Figure 3B) and that CXCR3 expression on SARS-CoV-2–specific CD4^+^ T cells declined with age in children (Figure 3D).

### Correlation of pediatric spike B_Mem_ and humoral responses.

**Figure 9:**
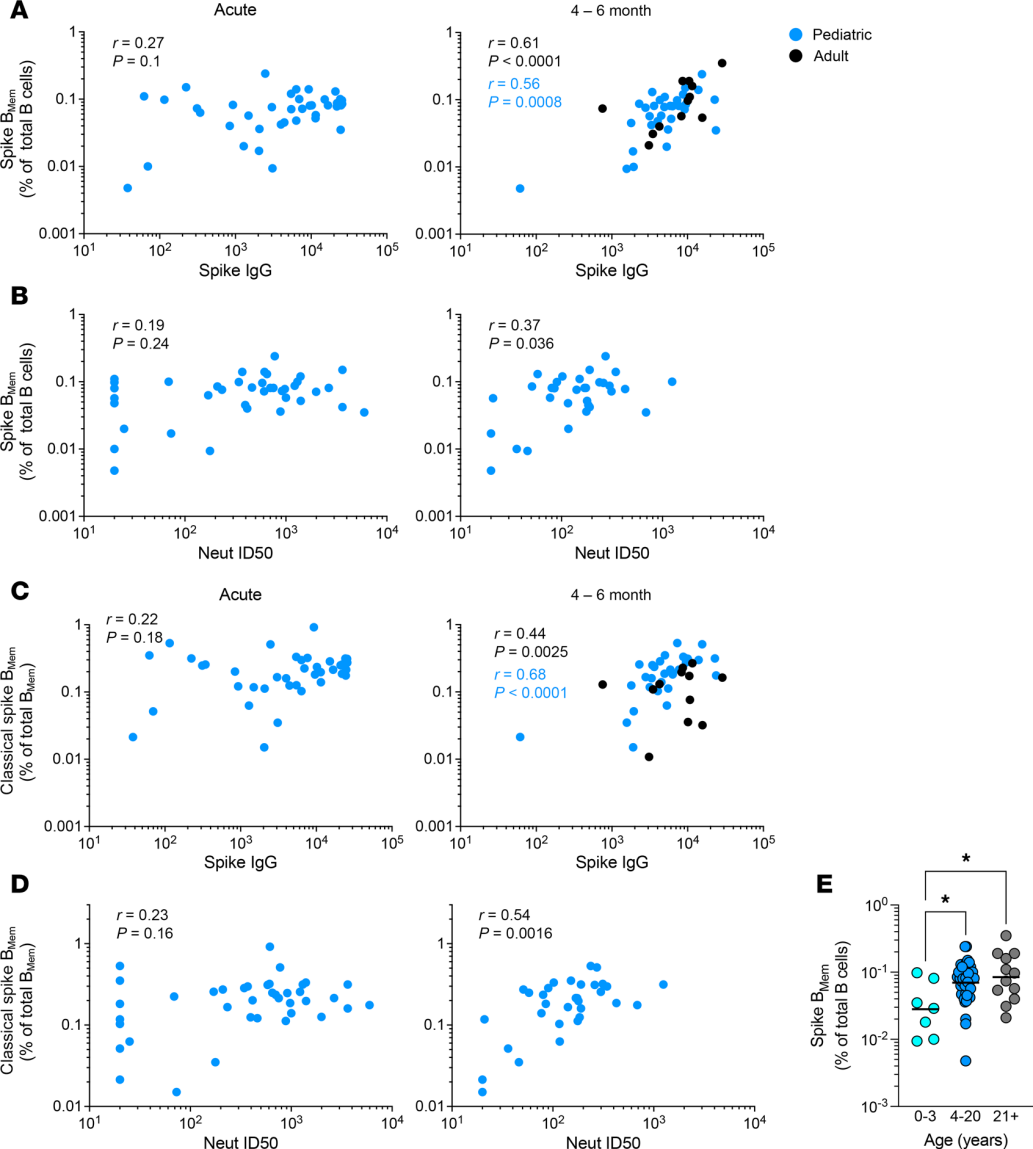
Correlation of pediatric spike B_Mem_ with humoral responses. (**A**) Correlation of spike IgG titers as determined by binding antibody multiplex assay (BAMA) at the acute (left) and 4- to 6-month (right) time points with spike B_Mem_ indicated as percentage of total B cells in children (blue) and adults (black). (**B**) Correlation of Neut ID_50_ titers at the acute (left) and 4- to 6-month (right) time points with spike B_Mem_ indicated as percentage of total B cells in children. (**C**) Correlation of spike IgG titers at the acute (left) and 4- to 6-month (right) time points with classical B_Mem_ indicated as percentage of total B_Mem_ in children (blue) and adults (black). (**D**) Correlation of Neut ID_50_ titers at the acute (left) and 4- to 6-month (right) time points with classical B_Mem_ indicated as percentage of total B_Mem_ in children. (**E**) Comparison of B_Mem_ responses in children less than 4 years old, older children, and adults. Center lines in **E** represent the GeoMean. In **A** and **C** 4- to 6-month time points, *r* values in black indicate Spearman correlation coefficient for the combined pediatric and adult data; *r* values in blue indicate Spearman correlation coefficient for the pediatric data alone. *P* values in **E** were calculated by Kruskal-Wallis test with Dunn’s correction, indicated as *P < 0.05.

We sought to determine how the distinct B_Mem_ responses in children correlated with circulating antibodies. Most serologic data from the BRAVE and MESSI studies were previously reported (18, 25), with additional data added for this study. Neither spike-specific IgG level nor viral neutralization was significantly correlated with the frequency of spike-specific B_Mem_ at the acute time point of SARS-CoV-2 infection (Figure 9, A and B). In contrast, both spike-specific IgG levels and viral neutralization were significantly correlated with the frequency of spike-specific B_Mem_ at 4–6 months postinfection (Figure 9, A and B; spike IgG: *r* = 0.56, *P* = 0.0008; Neut ID_50_: *r* = 0.37, *P* = 0.036). The relationship between spike-specific IgG levels and spike-specific B_Mem_ at 4–6 months postinfection was preserved with the addition of adult data (*r* = 0.61, *P* < 0.0001). Similar findings were observed for classical-phenotype spike-specific B_Mem_ at the 4- to 6-month time point (Figure 9, C and D; spike IgG: *r* = 0.68, *P* < 0.0001; addition of adult data: *r* = 0.44, *P* = 0.0025; Neut ID_50_: *r* = 0.54, *P* = 0.0016). Notably, children less than 4 years old had significantly lower frequencies of spike-specific B_Mem_ than older children or adults (Figure 9E). Spike IgG titers of young children were comparable to older children and adults (18), despite their reduced B_Mem_ frequencies. Last, we compared the frequencies of SARS-CoV-2–specific cT_FH_ and total CD4^+^ T cells with the antibody responses; however, we did not observe any significant correlations between SARS-CoV-2–specific cT_FH_ frequencies and neutralizing or binding antibody responses to SARS-CoV-2 (Supplemental Figure 5, E and F). In summary, total and classical spike-specific B_Mem_ were correlated with spike IgG levels and viral neutralization at 4–6 months after SARS-CoV-2 infection.

## Discussion

Despite similar rates of infection across age groups, children develop milder disease than adults in response to SARS-CoV-2 infection (37, 38). Cellular and humoral immune responses to SARS-CoV-2 infection play a pivotal role in disease severity and protective immunity among adults (39), but few studies have examined adaptive immune responses to infection in children (20, 40–42). Assessing adaptive immune responses to SARS-CoV-2 in children is important not only to understand how T and B cells contribute to pediatric immunity against SARS-CoV-2 but also to better understand the unique features of pediatric adaptive immune responses more broadly. In this study, we present a detailed analysis of virus-specific CD4^+^ T cell and B_Mem_ responses in 329 PBMC samples collected from a unique cohort of donors that include nonhospitalized, SARS-CoV-2–infected children and adolescents and compare the responses with those of mildly symptomatic adults. We show that children 4 years and older develop a robust, multifunctional SARS-CoV-2–specific CD4^+^ T cell response that generally peaks earlier than adult responses. Further, the pediatric acute CD4^+^ T cell response is skewed toward non-spike antigens, and higher non-spike/spike CD4^+^ T cell ratios are associated with asymptomatic infection. In addition, children have a higher frequency of CXCR3^+^ virus-specific CD4^+^ T cells than adults, consistent with a strong IFN-γ response during acute infection (43, 44). In contrast with older children, CD4^+^ T cell and cT_FH_ responses in children less than 4 years old were largely below the LOQ, and B_Mem_ responses were also lower in this age group. Despite this, children across the age spectrum mounted robust antibody responses with increased viral neutralization over time, suggestive of ongoing affinity maturation. Although the frequencies of total SARS-CoV-2–specific B_Mem_ were similar between children and adults, pediatric B_Mem_ skewed toward classical memory phenotypes and were more likely to express CXCR3, similar to pediatric CD4^+^ T cells. In children, B_Mem_ frequencies strongly correlated with persistence of spike-specific IgG and viral neutralization.

Most previous studies of T cell responses in children with mildly symptomatic COVID-19 have been limited by either small sample size or a lack of samples during acute infection (20, 40, 41), which may underlie conflicting reports regarding the magnitude and kinetics of the T cell response to SARS-CoV-2 infection (20, 22, 23, 45). We observed that most children develop robust and rapid T cell responses during acute infection. The kinetics of this response are consistent with trends seen in a recent study that included a small number of children with mildly symptomatic COVID-19 (40). Early induction of T cell responses in children may promote protection from severe disease, as early T cell responses in adults are associated with less severe COVID-19 (10, 12, 15, 46). Potential causes for the early T cell response in children are likely multifactorial. Children have preactivated type I and type III IFN responses in upper airway and lungs (3, 4, 44, 47, 48), which in addition to early suppression of viral replication in the respiratory tract may enable optimal activation of the innate immune system, resulting in enhanced or faster antigen presentation and earlier T cell activation (49–51).

We show that SARS-CoV-2–specific CD4^+^ T cells during acute infection are biased toward non-spike epitopes in children relative to adults. The higher non-spike/spike CD4^+^ T cell ratio in children reported here agrees with the ORF1ab/structural protein–specific T cell ratio previously observed in a pediatric SARS-CoV-2 infection cohort (20). Additionally, we show that a higher ratio of non-spike/spike CD4^+^ T cells was associated with asymptomatic disease, suggesting a protective role of non-spike-targeting CD4^+^ T cells. A similar association was previously reported in a small cohort of SARS-CoV-2–infected adults (15). Interestingly, ORF1ab epitopes are relatively more conserved and have been implicated in crossreactivity to previously endemic coronaviruses (32); however, we did not observe high levels of non-spike–specific T cells in uninfected children. These findings have implications for vaccine design in the context of viral evolution and the emergence of new variants given that non-spike epitopes are more conserved across variants (52).

In addition to distinct T cell response kinetics, we observed that children have fewer SARS-CoV-2–specific memory CD4^+^ T cells compared with adults. Several recent studies comparing immune responses in adults and children similarly found that virus-specific memory CD4^+^ T cells increased with age. In contrast, Dowell and colleagues found that children mount a stronger adaptive immune response to SARS-CoV-2 compared with adults; however, this study did not evaluate the contribution of different T cell populations to the overall response (20–22, 42, 45). A plausible explanation for higher acute CD4^+^ T cells and reduced memory CD4^+^ T cells in pediatric COVID-19 could be that heightened early innate immune responses rapidly reduce viral load, leading to diminished antigen-driven T cell responses over time.

Children under 4 years of age produced distinctly low virus-specific CD4^+^ T cell, cT_FH_ cell, and B_Mem_ responses compared with older children and adults. We speculate that this could be due to (a) a less mature adaptive immune system in infants and young children, (b) a lower need for T cell and B cell responses because of other compensatory immune mechanisms (e.g., innate immune response in the respiratory tract), or (c) virus-specific CD4^+^ T cells and B_Mem_ preferentially extravasating from blood and homing to tissues at the primary site of infection. Children under 4 years of age mounted comparable IgG antibody responses to those of older children and adults, which is most consistent with model (c).

Our findings of lower CD4^+^ T cell and B_Mem_ responses to SARS-CoV-2 in children under 4 years of age is in agreement with a recent study from Manfroi et al. that compared CD4^+^ T cell and B_Mem_ responses in children less than 5 years old to older children and adults (42). Particular strengths of our study are the inclusion of markedly more pediatric cases, specific investigation of CD4^+^ T cell subsets including cT_FH_, and higher resolution characterization of B_Mem_ phenotypes that highlight important differences between adaptive immune responses of children and adults to SARS-CoV-2.

Interestingly, B_Mem_ phenotyping herein revealed a bias of pediatric B_Mem_ toward a classical phenotype, which was stronger among SARS-CoV-2–specific B_Mem_. This may reflect that children eliminate viral antigen faster and thus have less tendency to generate activated and atypical B_Mem_ (53, 54). Alternatively, this could indicate that children have intrinsically different B cell responses to respiratory viral infections favoring generation of classical B_Mem_. Others have observed a propensity toward nonclassical B_Mem_ in adult patients hospitalized with COVID-19 (55), and our data highlight that age also plays a prominent role in the quality of B_Mem_ responses to SARS-CoV-2. Importantly, the abundance of CXCR3 in pediatric B_Mem_ contrasts with the association of CXCR3 expression with activated and atypical B_Mem_ phenotypes in adults (36), suggesting that B_Mem_ responses and phenotypes in children may be biologically distinct. To our knowledge, these distinct phenotypic differences between B_Mem_ of children and adults have not been previously reported in humans. Whether this bias toward classical B_Mem_ explains how children less than 4 years old maintain robust humoral immune responses despite decreased frequencies of antigen-specific CD4^+^ T cells and B_Mem_ warrants further investigation.

Children also produced a decreased antigen-specific cT_FH_ response compared with adults at all time points analyzed. However, a higher frequency of non-antigen-specific PD-1^+^CXCR5^+^CD4^+^ cT_FH_ cells was observed in children during acute infection, indicating an activated state of cT_FH_ cells overall. Similar observations regarding total (non-antigen-specific) activated cT_FH_ cells being higher in children were reported by Cohen and colleagues (20); however, these differences may or may not reflect SARS-CoV-2–specific responses and warrant further investigation.

Our study had several strengths and limitations. Strengths include thorough profiling of several features and subsets of SARS-CoV-2–specific CD4^+^ T cells and B_Mem_, the relatively large size of the cohort, and cohort development beginning in spring 2020, prior to the widespread deployment of COVID-19 vaccines. A methodological strength of this study is the use of the hybrid AIM and ICS assay, which not only detects antigen-specific cells accurately and sensitively but also provides a measure of cellular function via assessment of cytokine secretion in a cell-specific manner. While AIM assays have been validated for specificity (27, 31, 56), in some cases, bystander activation of CCR6^+^ cells occurs (57). Additionally, analyzing CD4^+^ T cell and B_Mem_ responses in peripheral blood does not provide a full picture of the adaptive immune responses in other tissues (58, 59). Another limitation of this study was a lack of resolution among the non-spike epitopes tested. Given the sampling constraints for pediatric participants, it was not feasible to test the reactivity of T cells against peptide pools from individual non-spike proteins. Future studies will be needed to identify the specific epitopes to which these responses were directed. Finally, we recognize that although our pediatric cohort was notable for its overall size, some of our analyses necessarily included only a subset of the pediatric samples and were thus limited in their statistical power; similar limitations pertain to our adult cohort.

In conclusion, we demonstrate that children concurrently mount robust and rapid CD4^+^ T cell, B_Mem_, and antibody responses to primary SARS-CoV-2 infection. We have identified differences between SARS-CoV-2–specific CD4^+^ T cell responses in children and adults, including the magnitude, kinetics, functionality, and dominant targets of these responses. Further, we identify associations between CD4^+^ T cell, B_Mem_, and humoral immune responses. Our findings suggest that differences in CD4^+^ T cell responses between children and adults could underlie observed differences in COVID-19 severity. They also identify how pediatric B_Mem_ responses to SARS-CoV-2 may be functionally distinct from those in adults. These findings highlight age-based immunological differences that enrich our understanding of protective immunity against SARS-CoV-2 and other respiratory viral pathogens.

## Methods

### Sex as a biological variable.

Our study involved both male and female human participants and considered sex as a biological variable.

### Study design and procedures.

The Duke BRAVE Kids study (25) was a prospective cohort study of children and adolescents (<21 years of age) with confirmed SARS-CoV-2 infection or close contact with an individual with confirmed SARS-CoV-2 infection. SARS-CoV-2 was detected from nasopharyngeal or nasal swabs through PCR testing performed for clinical care or through a quantitative real-time PCR assay, as previously described (25). Adult participants were enrolled in the MESSI (26). Participants in this prospective cohort were identified via enrollment in the community or through review of testing performed within the Duke University Health System (DUHS) or the Durham Veterans Affairs Health System (DVAHS). Testing for SARS-CoV-2 infection by PCR was performed either at the North Carolina State Laboratory of Public Health or through the clinical laboratories at either DUHS or DVAHS. Samples used in this study were from participants who were diagnosed with or exposed to SARS-CoV-2 between April 2020 and June 2021. The majority (all but 8) of participants were enrolled by December 2020. We collected exposure, sociodemographic, and clinical data at the time of enrollment through review of electronic medical records and a participant/caregiver questionnaire, including prior history of SARS-CoV-2 infection and receipt of SARS-CoV-2 vaccines; however, no study participant included in this study had received a vaccine at the time of sampling, determined both by chart review and patient report. For both children and adults, mild infection was defined as any PCR-confirmed infection for which the individual did not require hospitalization but experienced symptoms such as fever, cough, rhinorrhea, congestion, or diarrhea. Asymptomatic infection in children was defined as any PCR-confirmed infection for which the individual did not have any signs or symptoms of disease. Samples were obtained through home visits to collect whole blood from participants via venipuncture and to obtain other biospecimens. Serum was isolated from whole blood via centrifugation and frozen to –80°C before analysis. PBMCs were isolated from whole blood using density gradient centrifugation and stored in liquid nitrogen, as previously described (60). Follow-up visits were conducted at home or at a research clinic site approximately 2 and 6 months after acute infection.

### Combined AIM and ICS assay.

A combined AIM and ICS assay was used for the detection of SARS-CoV-2–specific T cells as described previously (61). PBMCs were plated in a 96-well, U-bottom plate at 1 × 10^6^ cells per well in RPMI media supplemented with 5% human AB serum (Gemini Bioproducts). PBMCs were incubated with 0.5 μg/mL of anti-CD40 mAb (Miltenyi Biotec) and fluorescently conjugated chemokine receptor antibodies (CXCR5, CXCR3, CCR7, CCR6) for 15 minutes. Following the 15-minute incubation, PBMCs were stimulated with 1 μg/mL of SARS-CoV-2 spike megapool (MP) containing overlapping peptides spanning the entire spike protein sequence (32) or CD4RE MP containing experimentally defined non-spike epitopes (33) for 24 hours at 37°C. PBMCs were also incubated with an equimolar amount of DMSO as a negative control or with 1 μg/mL staphylococcal enterotoxin B (SEB) as a positive control.

After 24 hours, Golgi-Plug and Golgi-Stop (BD Biosciences) along with AIM marker antibodies (OX40, CD40L, 4-1BB, CD69, PD-1, and ICOS) were added and incubated for an additional 4 hours. Supernatants were then collected and stored at –80°C for multiplexed quantification of cytokines. Cells were washed, Fc receptor–blocked (BioLegend), and surface-stained (CD3, CD38, CD8, CD14, CD16, CD20, CD45RA, CD27, CD4, LIVE/DEAD) for 30 minutes at 4°C in the dark. Cells were washed, fixed with 4% formaldehyde for 10 minutes at 4°C, and permeabilized and blocked with 10% human AB serum in saponin buffer (Sigma-Aldrich) for 5 minutes. Cells were then intracellularly stained (TNF, IL-2, IL-17, GzmB, IL-10, and IFN-γ) for 30 minutes at 4°C and acquired on a Cytek Aurora. Additional information on antibodies can be found in Supplemental Table 3.

PBMC samples below 80% viability or with an AIM^+^ response less than half of the median SEB response for all samples were eliminated from downstream analysis. AIM^+^ flow cytometry gates were set to a maximum DMSO signal of 0.1 across all samples. Samples with a DMSO signal of 0 were arbitrarily assigned a value of 0.005 to allow calculation of the GeoMean. For samples with 2 DMSO replicates, the GeoMean of the replicates was calculated as the background signal for that sample; for samples with 1 DMSO replicate, the individual value was used as background signal. For each sample, antigen-specific CD4^+^ T cell responses were background-corrected by subtracting the DMSO negative control values from the raw values for spike or non-spike AIM^+^ responses. The lower LOQ was set to be the GeoMean of all DMSO replicates. The LOD was defined as LOQ/2. A stimulation index (SI) for each sample was calculated as the background-subtracted signal for spike or non-spike responses divided by the GeoMean DMSO signal for that sample. Samples with an SI less than 2 were considered nonresponders and were set to the LOD.

### B_Mem_ flow cytometry assay.

To detect SARS-CoV-2–specific B_Mem_, biotinylated full-length spike (Acro Biosystems, SPN-C82E9), RBD (BioLegend, 793904), or full-length nucleocapsid (Sino Biological, 40588-V27B-B) was tetramerized to fluorescently conjugated streptavidin as previously described (62). Biotinylated spike was mixed with BV421 (BioLegend, 405226) or Alexa Fluor 647 (Thermo Fisher Scientific, S21374) at a 10:1 ratio (4:1 molar ratio). Biotinylated RBD was mixed with BV711 (BioLegend, 405241) or PE-Cy7 (BioLegend, 405206) at a 2.2:1 ratio (4:1 molar ratio). Biotinylated nucleocapsid was mixed with BV605 (BioLegend, 405229) or BV785 (BioLegend, 405249) at a 5.5:1 ratio (6:1 molar ratio). All streptavidin was added in a stepwise addition by adding 1/3 of streptavidin to the biotinylated protein at a time and incubating for 15 minutes in between. PBMCs were plated in a 96-well, U-bottom plate at up to 10 × 10^6^ cells per well, stained with 1:20 Fc block (BioLegend, 422302), washed with FACS buffer (2% FBS in PBS), and incubated with 5 μM free d-biotin (Avidity, Bir500A), 150 ng per spike probe (300 ng total), 16.4 ng per RBD probe (32.8 ng total), 40 ng per nucleocapsid probe (80 ng total), and 20 ng streptavidin PE-Cy5.5 (Thermo Fisher Scientific, SA1018) in Brilliant Buffer (BD Biosciences, 566349) for 1 hour at 4°C in the dark. Streptavidin PE-Cy5.5 was used as an empty probe to remove streptavidin-binding B cells from the analysis. Following the 1-hour incubation, PBMCs were washed with FACS buffer and surface-stained with antibodies for 30 minutes at 4°C. PBMCs were then washed with FACS buffer, stained with 1:200 Live Dead Blue (Thermo Fisher Scientific, L34962) diluted in PBS, incubated for 30 minutes at 4°C, washed again, and acquired on an Aurora. The limit of sensitivity was calculated as the median + 2 times the SD of the SARS-CoV-2–unexposed donors. Donors with fewer than 10 SARS-CoV-2–specific B_Mem_ were excluded from any phenotyping analysis (summary statistics for the samples in this analysis can be found in Supplemental Table 4). Additional information on antibodies can be found in Supplemental Table 5.

### Cytokine bead assay.

Cytokine profiling was performed on top CD4^+^AIM^+^ (CD40L^+^OX40^+^) responders using a LEGENDplex HU Th Cytokine Panel (12-plex) (BioLegend, 741027) for the detection of IL-5, IL-13, IL-2, IL-6, IL-9, IL-10, IFN-γ, TNF-α, IL-17A, IL-17F, IL-4, and IL-22. Supernatants were thawed, centrifuged at 1,000*g* for 5 minutes, and run undiluted using the filter plate method following the manufacturer’s instructions. Samples were acquired on an Aurora, and data were analyzed using the LEGENDplex Data Analysis Software Suite.

Antigen-specific responses were background-subtracted using values from the DMSO-negative controls. LOD was calculated as the concentration of the blank plus 3 times the SD. LOQ was calculated as the concentration of the blank plus 10 times the SD. The lowest value was set to the LOD for each cytokine measured.

### Measurement of SARS-CoV-2–specific antibodies in serum.

SARS-CoV-2–specific antibodies were assessed using a BAMA, as previously described (18). Briefly, we evaluated IgG binding to the following antigens: whole spike (Sino Biological, 40589-V08B1), S1 (Sino Biological, 40591-V08H), S2 (Sino Biological, 40590-V08B), RBD (Sino Biological, 40592-V08H), NTD (Sino Biological, 40591-V49H), NC (Sino Biological, 40588-V08B), and M (MyBiosource, MBS8574735) proteins. Antigens were covalently coupled to magnetic fluorescent beads (MagPlex biospheres, Luminex). Unconjugated (blank) beads were included to monitor nonspecific binding. Antigen-coupled beads were incubated with a 1:400 serum dilution for measurement of IgG. Antibody binding to the bead-coupled antigens was then detected with PE-conjugated mouse anti-human IgG (Southern Biotech, 9040-09) using a Bio-Plex 200 instrument (Bio-Rad Laboratories), which rendered an MFI for each sample. The assay positivity threshold (mean MFI plus 3 SDs) was defined based on readings from 10 sera samples collected between 2013 and 2014, prior to the COVID-19 pandemic.

### SARS-CoV-2 pseudovirus neutralization assays.

SARS-CoV-2 neutralization was measured with spike-pseudotyped viruses in HEK293T-hACE2 cells (BEI Resources) as a function of reduction in Luc reporter activity, as previously described (18). Briefly, we used an envelope-deficient HIV-based lentiviral system to produce viral particles pseudotyped with the SARS-CoV-2 D614G variant spike. Pseudovirions were produced in HEK293T/17 cells (ATCC), which were transfected with spike plasmid, lentiviral backbone plasmid (pCMV ΔR8.2), a firefly Luciferase reporter gene plasmid (pHR′ CMV Luc), and a TMPRSS2-expressing plasmid. Neutralization assays were performed using heat-inactivated serum samples that were serially diluted 5-fold to 8 points in duplicate and incubated with pseudovirus for 1–1.5 hours at 37°C in 96-well, flat-bottom, poly-l-lysine–coated culture plates (Corning Biocoat). HEK293T-hACE2 cells were suspended using TrypLE Select Enzyme solution (Thermo Fisher Scientific) and immediately added to all wells (10,000 cells in 100 μL of growth medium per well). Control wells included 8 wells that only received cells and virus (virus control) and 8 wells with cells only (background control). The serum, pseudovirus, and cells were incubated together for 72 hours, after which the medium was removed and 30 μL of Promega 1× lysis buffer was added to each well. After incubation for 10 minutes at room temperature, 100 μL Bright-Glo Luc reagent (Promega) was added to all wells. After an additional 2 minutes of incubation, 110 μL of the cell lysate from each well was transferred to a black/white plate. Luminescence was measured using a PerkinElmer Life Sciences model Victor3 luminometer. Neutralization titers represent the serum dilution at which relative luminescence units (RLUs) were reduced by either 50% (ID_50_) or 80% (ID_80_) compared with virus control wells after subtraction of background RLUs.

### Viral load estimation.

SARS-CoV-2 RNA copies per milliliter were determined using a 2-step real-time quantitative PCR assay developed in the Clinical Laboratory Improvement Amendments–certified Immunology and Virology Quality Assessment Center at the Duke Human Vaccine Institute. DSP Virus/Pathogen Midi Kits (QIAGEN) were used to extract viral RNA on a QIAsymphony SP automated sample preparation platform. A reverse primer specific to the SARS-CoV-2 envelope gene was annealed to the extracted RNA and reverse-transcribed into cDNA using SuperScript III Reverse Transcriptase and RNaseOut (Thermo Fisher Scientific). cDNA was treated with RNase H and then added to a custom 4× TaqMan Gene Expression Master Mix (Applied Biosystems) that contained envelope gene–specific primers and a fluorescently labeled hydrolysis probe; quantitative PCR was carried out on a QuantStudio 3 Real-Time PCR system (Thermo Fisher Scientific). SARS-CoV-2 RNA copies per reaction were interpolated using quantification cycle data and a serial dilution of a highly characterized custom DNA plasmid that contained the SARS-CoV-2 envelope gene sequence. The LOQ was 62 RNA copies/mL of sample as determined by an extensive validation process consistent for use in a clinical setting.

### Statistics.

All flow cytometry data were analyzed using FlowJo v.10 (BD Biosciences). Statistical analyses were performed in GraphPad Prism 10 (GraphPad Software). For all analyses, a 2-sided 5% type I error rate was used unless otherwise noted. *P* < 0.05 was considered statistically significant. Due to the constraints of obtaining human samples during a global pandemic, we employed a convenience sampling approach to derive our pediatric and adult cohorts. As such, not all data were available at all time points. For most analyses, either Mann-Whitney *U* tests for comparison of 2 groups or Kruskal-Wallis tests for comparison of 3 or more groups were used, unless otherwise noted; where relevant, Holm-Šídák (Mann-Whitney) or Dunn’s (Kruskal-Wallis) corrections for multiple comparisons were used, and all reported *P* values and representative asterisks are appropriately adjusted. Analyses such as those in Figure 1, B–D, and similar were designed to answer 3 distinct questions: (a) Are there differences across time points within the pediatric age group? (b) Are there differences across time points within the adult age group? (c) Are there differences at the same time point across the pediatric and adult age groups? As such, analyses (a) and (b) are performed with independent Kruskal-Wallis tests with Dunn’s correction, and analysis (c) is performed with multiple Mann-Whitney tests with Holm-Šídák correction. Additional details can be found in the relevant Methods subsections, Results, figures, and corresponding figure legends.

### Study approval.

The BRAVE Kids study and the MESSI study are being conducted within the DUHS in Raleigh-Durham, North Carolina, USA. DUHS is a large, integrated health system consisting of 3 hospitals and over 100 outpatient clinics. These studies were approved by the DUHS Institutional Review Board (Pro00106150 and Pro00100241). Informed consent was obtained from all study participants or their legal guardians with written approval obtained using an electronic consent document. Informed assent was also obtained for all children 8 years of age or older. All study protocols were conducted in accordance with the Declaration of Helsinki, applicable regulations, and local policies.

### Data availability.

All raw data files are available upon request. Supporting Data Values are provided with this paper in the supplement.

## Author contributions

SC, MSK, NB, and JHH designed the study. MSK, JHH, ATR, and NAT enrolled BRAVE Kids participants. CWW, TWB, EP, and MTM enrolled MESSI participants. NB and YD designed and optimized the hybrid AIM ICS assay. AM performed the hybrid AIM ICS and cytokine bead assay and the B cell flow cytometry. LBH and NB performed data analysis. AG, DW, and AS designed and validated the peptide MPs for the AIM ICS and cytokine bead assay. GGF and SRP generated and provided the antibody binding and neutralization data. LBH, NB, and SC wrote the manuscript with input from coauthors. The order of the co–first authors was based on the role of LBH in the final data analysis and preparation of the manuscript and figures.

## Supplementary Material

Supplemental data

ICMJE disclosure forms

Supporting data values

## Version 1 09/04/2025

In-Press Preview

## Version 2 10/22/2025

Electronic publication
