# Supplementary material for: Pediatric T cell and B cell responses to SARS-CoV-2 infection
Source: JCI Insight. 2025 Sep 4;10(20):e196032. doi: 10.1172/jci.insight.196032 (PMC12581660; doi:10.1172/jci.insight.196032)
Supplement: Supplemental data [file jciinsight-10-196032-s041.pdf]

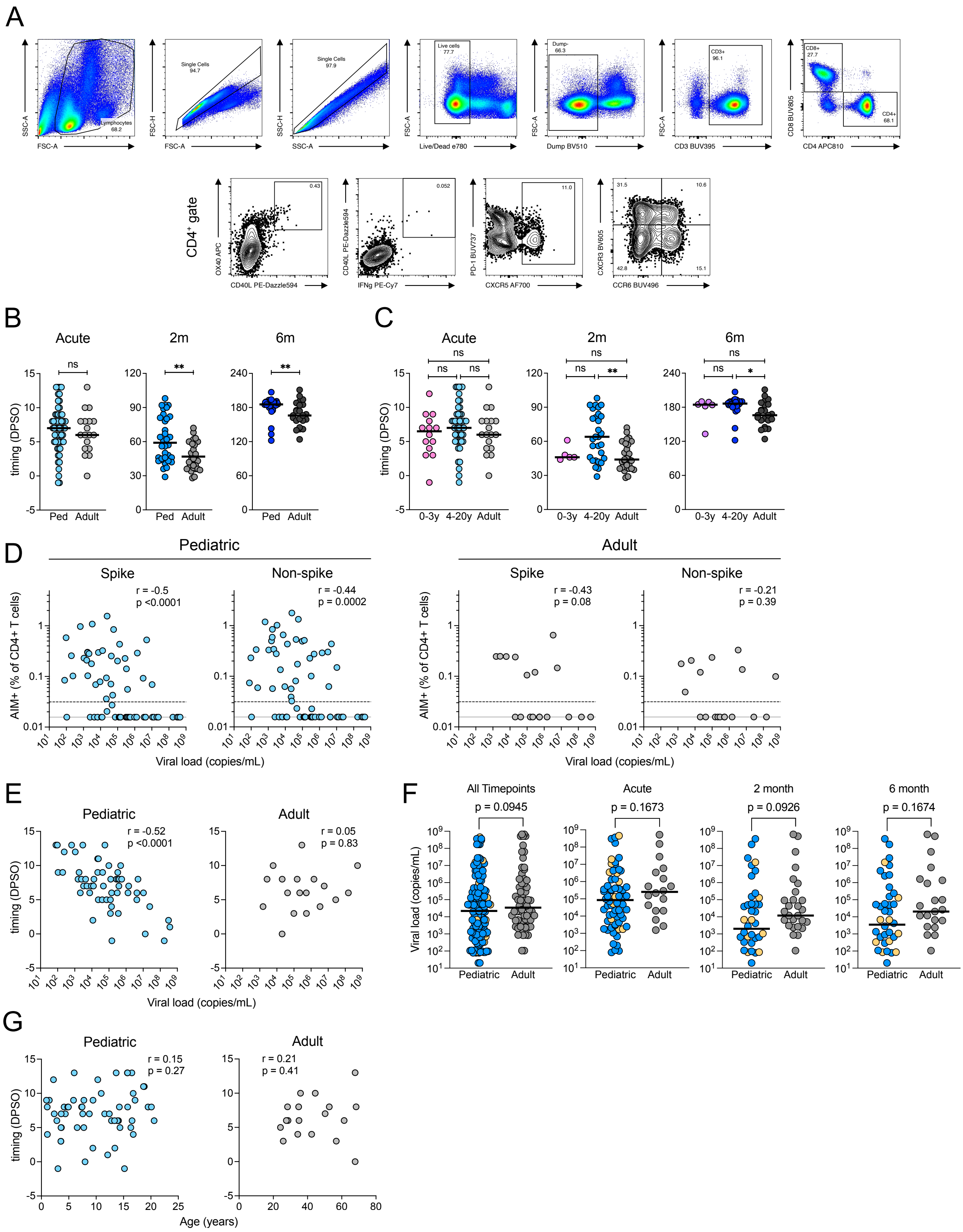

Supplemental Figure 1. Gating strategy for flow cytometry analysis, viral load and sample collection times

(**A**) Gating strategy for hybrid AIM and ICS analysis of SARS-CoV-2-specific CD4+ T cells. (**B** and **C**) Blood sample collection times indicated as days post symptom onset (DPSO) at the acute, 2-month ("2m"), and 6-month ("6m") timepoints. (**D**) Correlation between spike- and non-spike-AIM+ response in children and adults (as indicated) with viral load at the acute timepoint. (**E**) Correlation between blood sample collection times indicated as days post symptom onset (DPSO) with viral load in children and adults (as indicated). (**F**) Comparison of viral loads between children and adults; orange circles represent asymptomatic children. (**G**) Correlation between blood sample collection times indicated as days post symptom onset (DPSO) with donor age in children and adults (as indicated). Centerlines in **B** and **C** represent the median. Dotted line in **D** indicates the limit of quantification of the assay determined by calculating the geomean of the background (DMSO treated); solid grey line indicates the lower limit of detection. "AIM$^+$" denotes the frequencies of OX40$^+$CD40L$^+$ cells as percentage of CD4+ T cells. "Ped" in **B** represents the pediatric group. 'P' values for **B** and **F** were calculated by Mann-Whitney test and for **C** by Kruskal-Wallis test with Dunn's correction, and are indicated as *p<0.05, **p<0.01, ***p<0.001, ****p<0.0001, ns=non-significant. 'r' in **D**, **E**, and **G** indicates Spearman correlation coefficient.

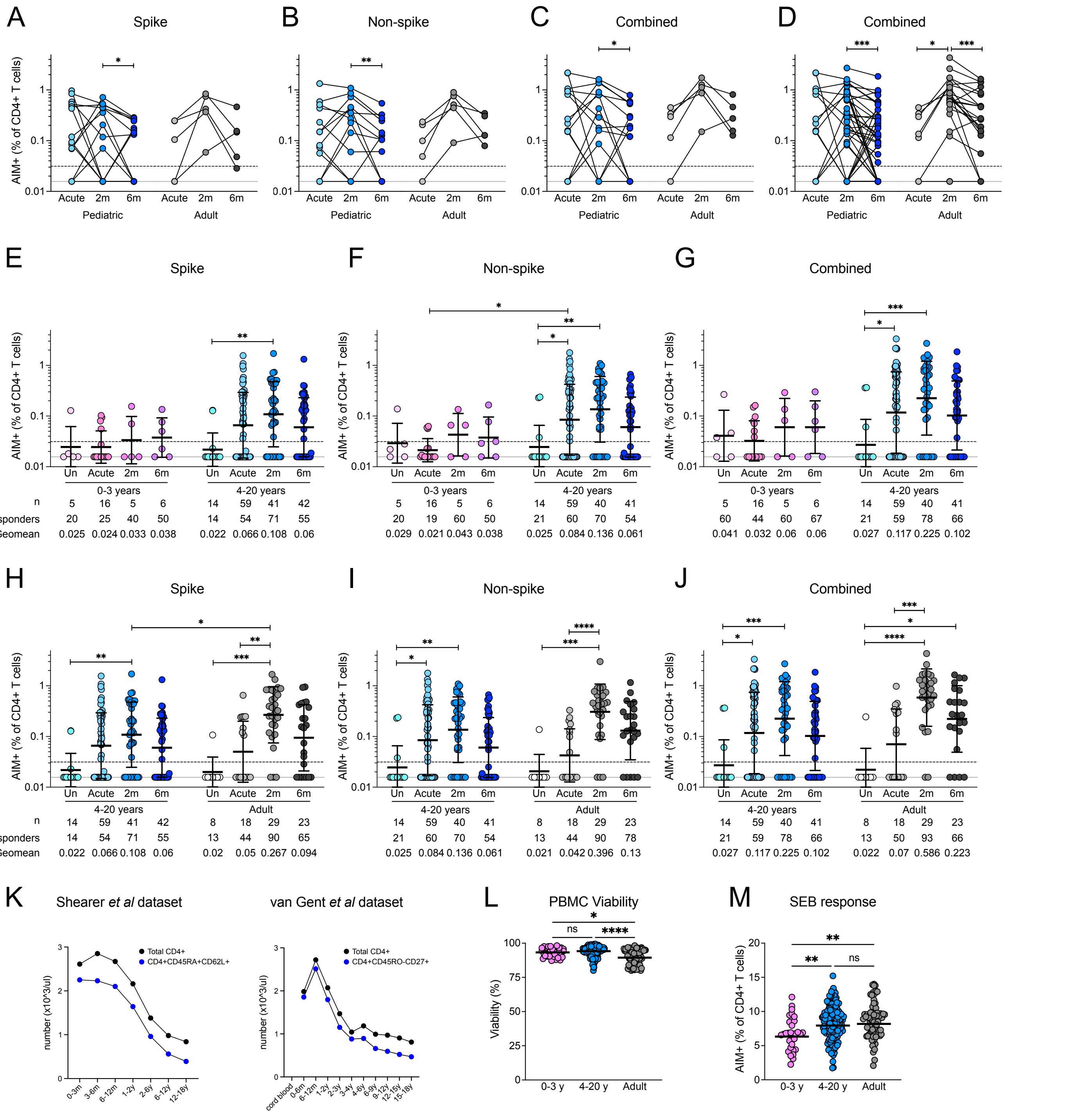

Supplemental Figure 2. SARS-CoV-2–specific CD4+ T cell response to SARS-CoV-2 increases with age

(**A-C**) Paired longitudinal (**A**) spike-specific, (**B**) non-spike specific, and (**C**) combined spike and non-spike-specific CD4+ T cell responses at indicated timepoints post-infection from donors that provided samples for all three timepoints. (**D**) Spike and non-spike combined CD4+ T cell responses at indicated timepoints post-infection. (**E-G**) Frequencies of SARS-CoV-2 (**E**) spike-specific, (**F**) non-spike-specific, and (**G**) combined spike- and non-spike-specific CD4+ T cells across indicated timepoints of infection in younger (0-3 years) and older (4-20 years) children. (**H-J**) Frequencies of SARS-CoV-2 (**H**) spike-specific, (**I**) non-spike-specific, and (**J**) combined spike- and non-spike- CD4+ T cells across indicated timepoints of infection in children and adults after removing the 0-3 age group from the pediatric cohort. (**K**) Data extracted from Shearer *et al* (DOI: 10.1016/j.jaci.2003.07.003) and van Gent *et al* (DOI: 10.1016/j.clim.2009.05.020) showing changes with age (months = "m", years = "y") in CD4+ T cell numbers per unit volume of blood. (**L**) Percent of viable PBMCs upon thawing, measured by a viability dye. (**M**) Frequencies of AIM^+ T cells from the indicated sample groups upon stimulation with SEB. Centerlines and error bars in **E-J** represent the geometric mean and geometric standard deviation, respectively. Centerlines in **L** and **M** represent the geometric mean. Dotted line in **A-J** indicates the limit of quantification of the assay determined by calculating the geomean of the background (DMSO treated); solid grey line indicates the lower limit of detection. Uninfected participants are indicated as "Un"; 2-month and 6-month timepoints as "2m" and "6m", respectively. "AIM^+" denotes the frequencies of $OX40^+CD40L^+$ cells as percentage of CD4+ T cells. 'P' values for **A-D** were calculated by Wilcoxon test, for **E-J** by Kruskal-Wallis test with Dunn's correction for comparisons within the pediatric and adult age groups and by Mann-Whitney test with Holm-Sidak correction for comparisons across the pediatric and adult age groups, and for **L** and **M** by Kruskal-Wallis test with Dunn's correction, and are indicated as *p<0.05, **p<0.01, ***p<0.001, ****p<0.0001; non-significant 'p' values are not shown or are indicated as 'ns' **L** and **M**.

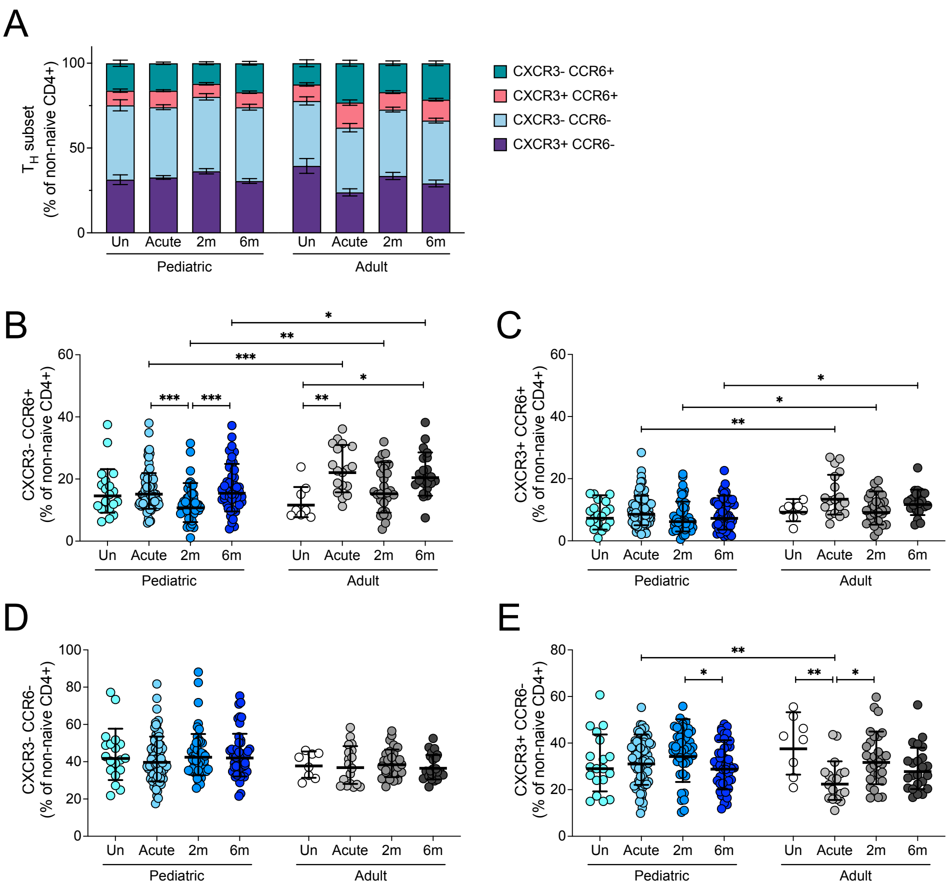

Supplemental Figure 3. T helper subset distribution in SARS-CoV-2 infected children and adults

(**A**) Proportion plot showing distribution of T helper subsets among total non-naïve CD4+ T cells in children and adults at indicated stages of SARS-CoV-2 infection. (**B-E**) Percentage of CXCR3$^-$CCR6$^+$ (**B**), CXCR3$^+$CCR6$^+$ (**C**), CXCR3$^-$CCR6$^-$ (**D**), and CXCR3$^+$CCR6$^-$ (**E**) cells among total non-naïve CD4+ T cells across indicated time points post-infection in children and adults. Centerlines and error bars in **B-E** represent the geometric mean and geometric standard deviation, respectively. Uninfected participants in **A-E** are indicated as "Un"; 2-month and 6-month timepoints as "2m" and "6m", respectively. 'P' values were calculated for **B-E** by Kruskal-Wallis test with Dunn's correction for comparisons within the pediatric and adult age groups and by Mann-Whitney test with Holm-Sidak correction for comparisons across the pediatric and adult age groups and are indicated as *p<0.05, **p<0.01, ***p<0.001, ****p<0.0001; non-significant 'p' values are not shown.

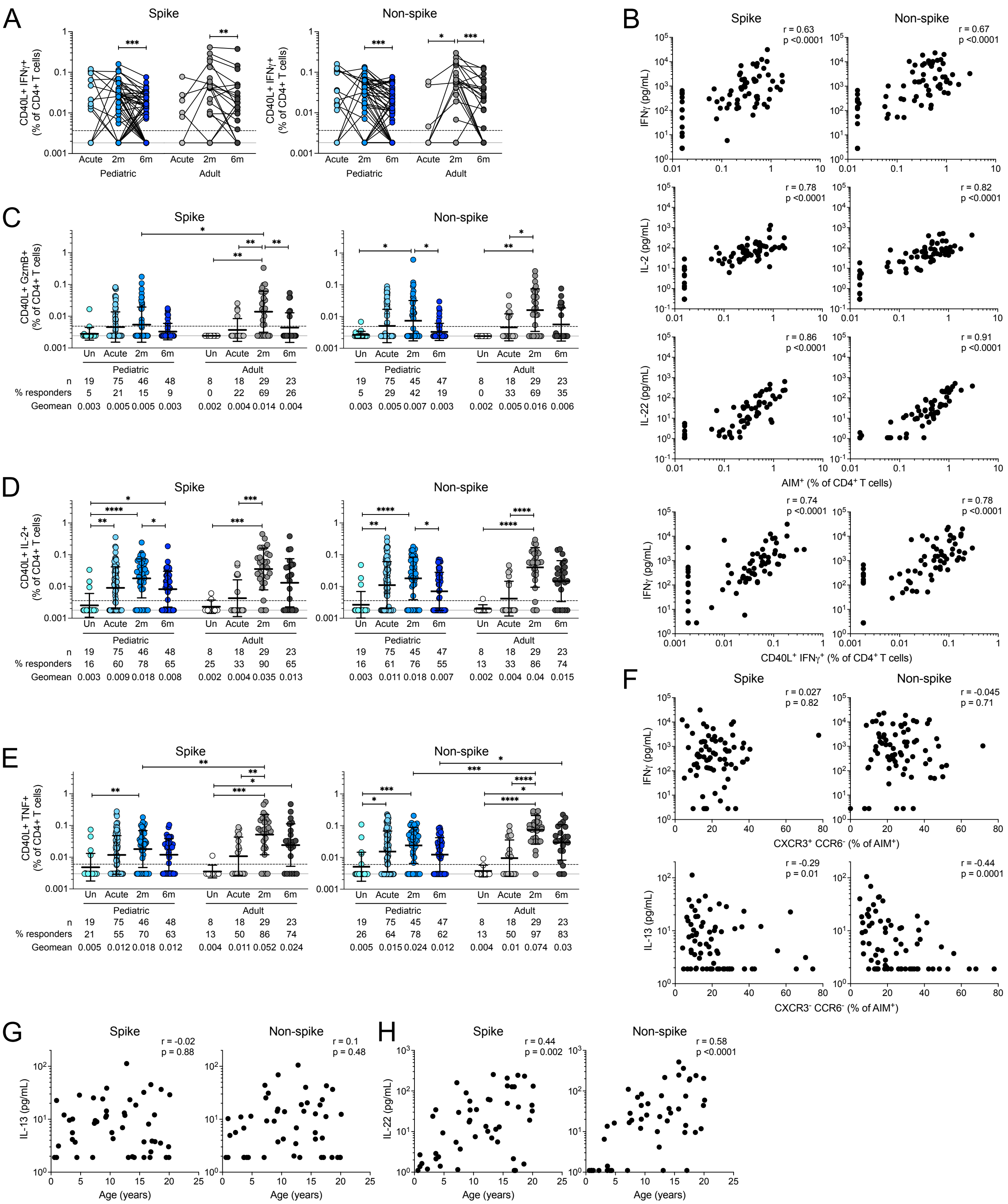

Supplemental Figure 4. SARS-CoV-2-specific CD4+ T cell cytokine response to SARS-CoV-2 in children

(**A**) Paired longitudinal spike- (left) and non-spike- (right) specific IFNγ producing CD4+ T cell frequencies at indicated time points post-infection from donors that provided samples for two or three time points. (**B**) Correlation between the indicated secreted cytokines measured by CBA and the frequency of OX40$^+$CD4+0$^+$ CD4+ T cells (top six panels) or CD40L$^+$IFNγ$^+$ CD4+ T cells (bottom two panels) measured by AIM ICS assay. (**C-E**) Frequencies of SARS-CoV-2 spike- (left) and non-spike- (right) specific, GzmB-producing (**C**), IL-2-producing (**D**), and TNF-producing (**E**) CD4+ T cells across indicated time points of infection in children and adults. (**F**) Correlation between secreted cytokine measured by CBA and the frequency of T helper subset determined by hybrid AIM assay. Indicated populations shown as percent of OX40$^+$CD40L$^+$CD4+. (**G**) Correlations of secreted IL-13 by spike- (left) and non-spike- (right) specific T cells at the acute phase of infection with age. (**H**) Correlations of secreted IL-22 by spike- (left) and non-spike- (right) specific T cells at the acute phase of infection with age. Centerlines and error bars in **C-E** represent the geometric mean and geometric standard deviation, respectively. Dotted line in **A** and **C-E** indicates the limit of quantification of the assay determined by calculating the geomean of the background (DMSO treated); solid grey line indicates the lower limit of detection. Uninfected participants are indicated as "Un"; 2-month and 6-month timepoints as "2m" and "6m", respectively. "AIM$^+$" in **B** and **F** denotes OX40$^+$CD40L$^+$ CD4+ T cells. 'P' values for **A** were calculated by Wilcoxon test, for **C-E** by Kruskal-Wallis test with Dunn's correction for comparisons within the pediatric and adult age groups and by Mann-Whitney test with Holm-Sidak correction for comparisons across the pediatric and adult age groups; and are indicated as *p<0.05, **p<0.01, ***p<0.001, ****p<0.0001; non-significant 'p' values are not shown. 'r' in **B** and **F-H** indicates Spearman correlation coefficient.

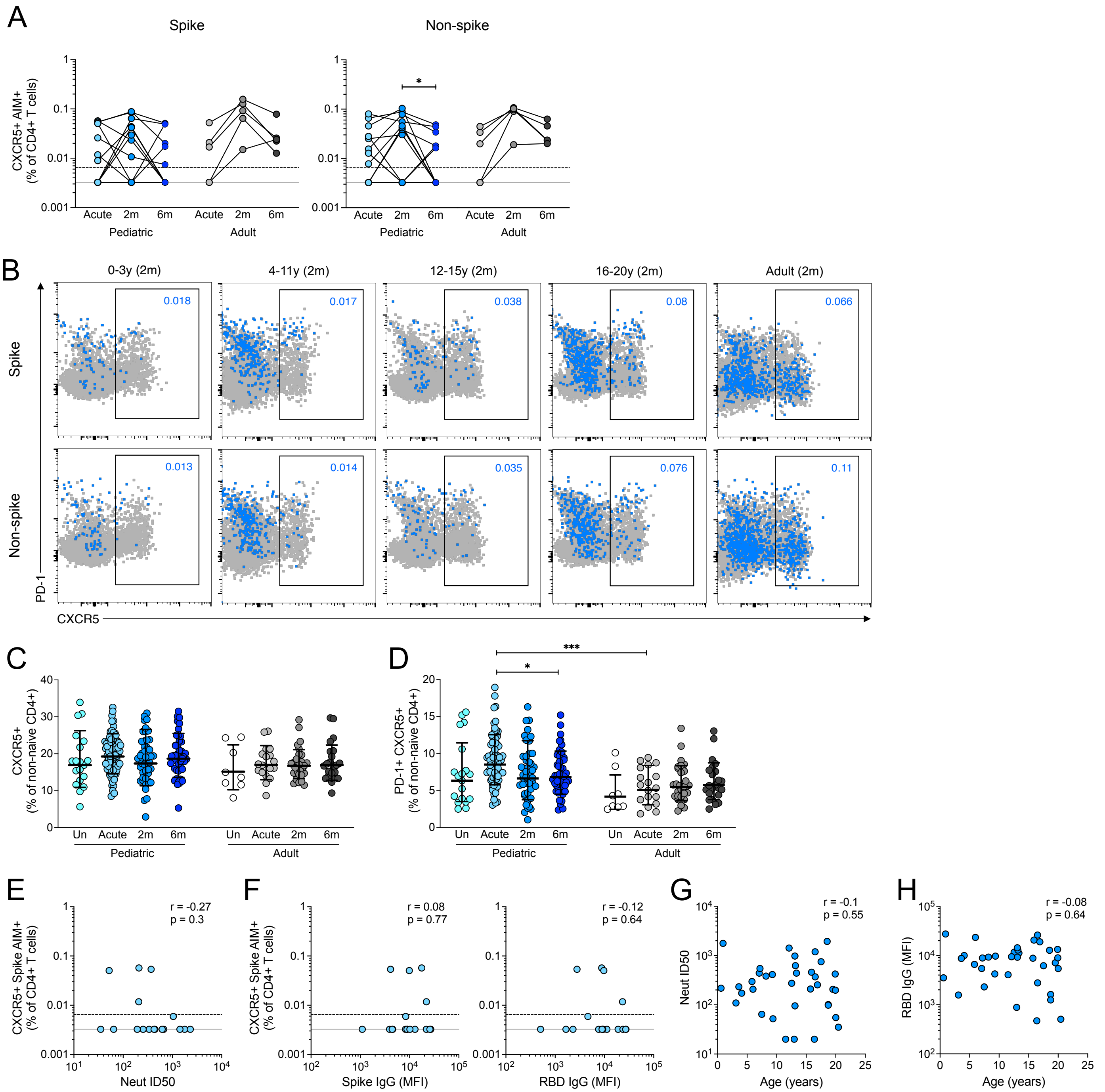

Supplemental Figure 5. cT$_{FH}$ response in SARS-CoV-2 infected children and adults

(**A**) Paired longitudinal spike- (left), non-spike- (right) specific cT$_{FH}$ frequencies at indicated timepoints post-infection from donors that provided samples for all three time points. (**B**) Representative flow cytometry plots from the indicated age-groups showing SARS-CoV-2 spike- (top) and non-spike- (bottom) specific AIM$^+$ CD4+ T cells (blue) overlayed on total CD4+ T cells (grey) with the CXCR5$^+$ gate representing cT$_{FH}$ cells. Numbers in blue indicate the frequency of SARS-CoV-2 specific cT$_{FH}$ amongst total CD4+ cells. (**C**) Frequencies of total cT$_{FH}$ cells among total non-naïve CD4+ T cells in children and adults at indicated time points. (**D**) Frequencies of PD1$^+$ CXCR5$^+$ cT$_{FH}$ cells among total non-naïve CD4+ T cells in children and adults at indicated time points. (**E**) Correlation of Spike-specific cT$_{FH}$ frequencies at the acute phase of infection plotted against neutralizing antibody measured during the convalescent phase using a pseudovirus (614G) assay shown as 50% inhibitory dilution. (**F**) Correlation of Spike-specific cT$_{FH}$ frequencies at the acute phase of infection with indicated antigen binding antibody levels measured 2 months post-infection using a Luminex based multiplex assay. (**G**) Correlation of neutralizing antibody titers at 50% inhibitory dilution measured 2 months post-infection with age. (**H**) Correlation of SARS-CoV-2 RBD-binding antibody levels measured 2 months post-infection with age. Centerlines and error bars in **C** and **D** represent the geometric mean and geometric standard deviation, respectively. Dotted line in **A**, **E**, and **F** indicates the limit of quantification of the assay determined by calculating the geomean of the background (DMSO treated); solid grey line indicates the lower limit of detection. Uninfected participants are indicated as "Un"; 2-month and 6-month timepoints as "2m" and "6m", respectively. "AIM$^+$" denotes the frequencies of OX40$^+$CD40L$^+$ cells amongst total CD4+ T cells. 'P' values were calculated for **A** by Wilcoxon test, for (C-D) by Kruskal-Wallis test with Dunn's correction for comparisons within the pediatric and adult age groups and by Mann-Whitney test with Holm-Sidak correction for comparisons across the pediatric and adult age groups, and are indicated as *p<0.05, **p<0.01, ***p<0.001, ****p<0.0001; non-significant 'p' values are not shown. 'r' in (E-H) indicates Spearman correlation coefficient.

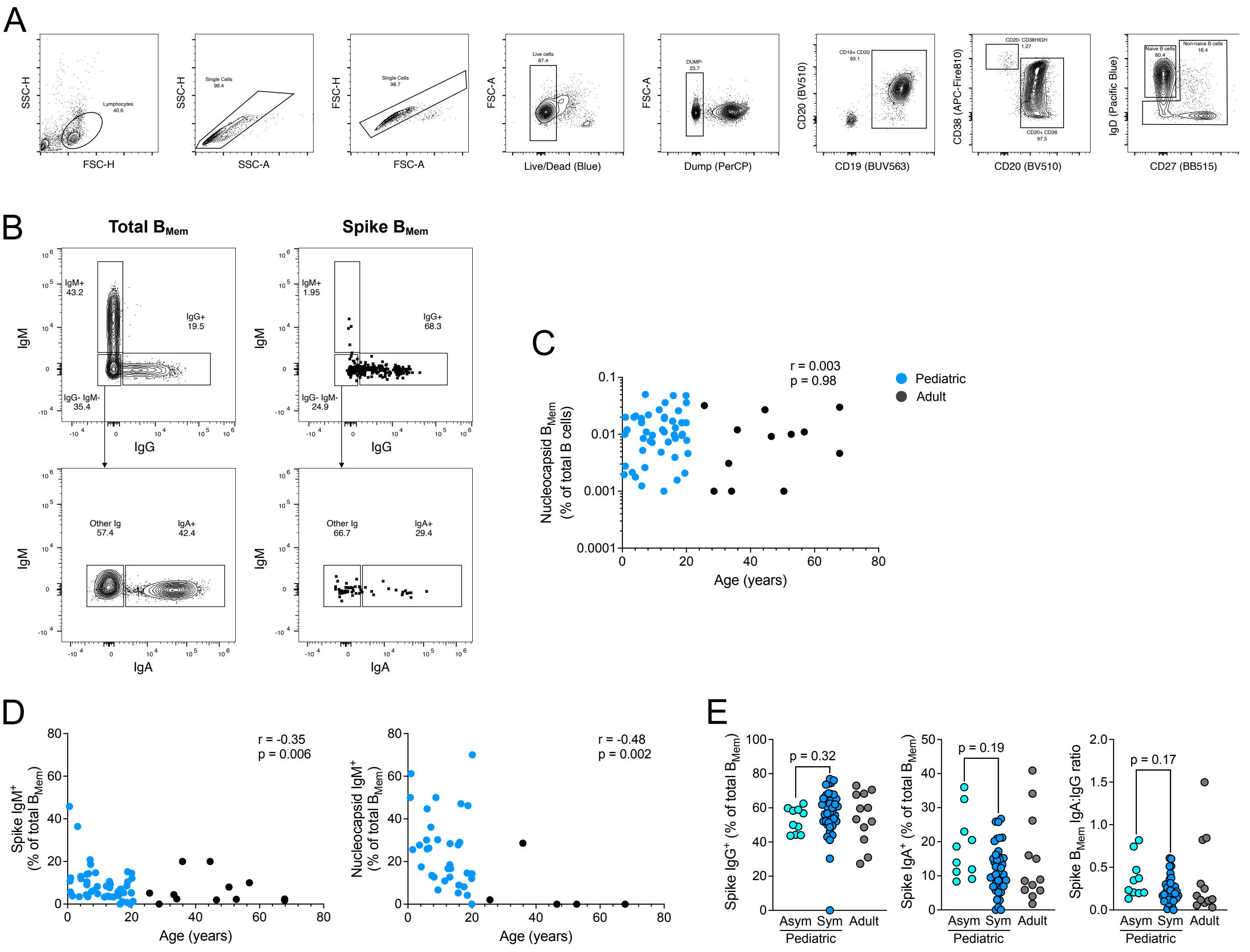

Supplemental Figure 6. Isotype distribution of SARS-CoV-2-specific memory B cells in children and adults

(**A**) Gating strategy for SARS-CoV-2-specific memory B cell analysis (additional representative gates are shown in their respective figures). (**B**) Representative flow cytometry plots showing Ig isotypes among total memory B cells (left) and spike-specific memory B cells (right). (**C**) Correlation of Nucleocapsid binding memory B cell frequencies with age in the pediatric group. (**D**) Correlations of IgM+ memory B cells as percent of Spike (left) and Nucleocapsid (right) binding memory B cells with age. (**E**) Frequencies of Spike Ig isotypes in asymptomatic ('Asym') or symptomatic ('Sym') pediatric or adult participants. n=50 in the pediatric group; n=12 in the adult group. 'P' values for **E** were calculated by by Kruskal-Wallis test with Dunn's correction. 'r' in **C** and **D** indicates Spearman correlation coefficient.

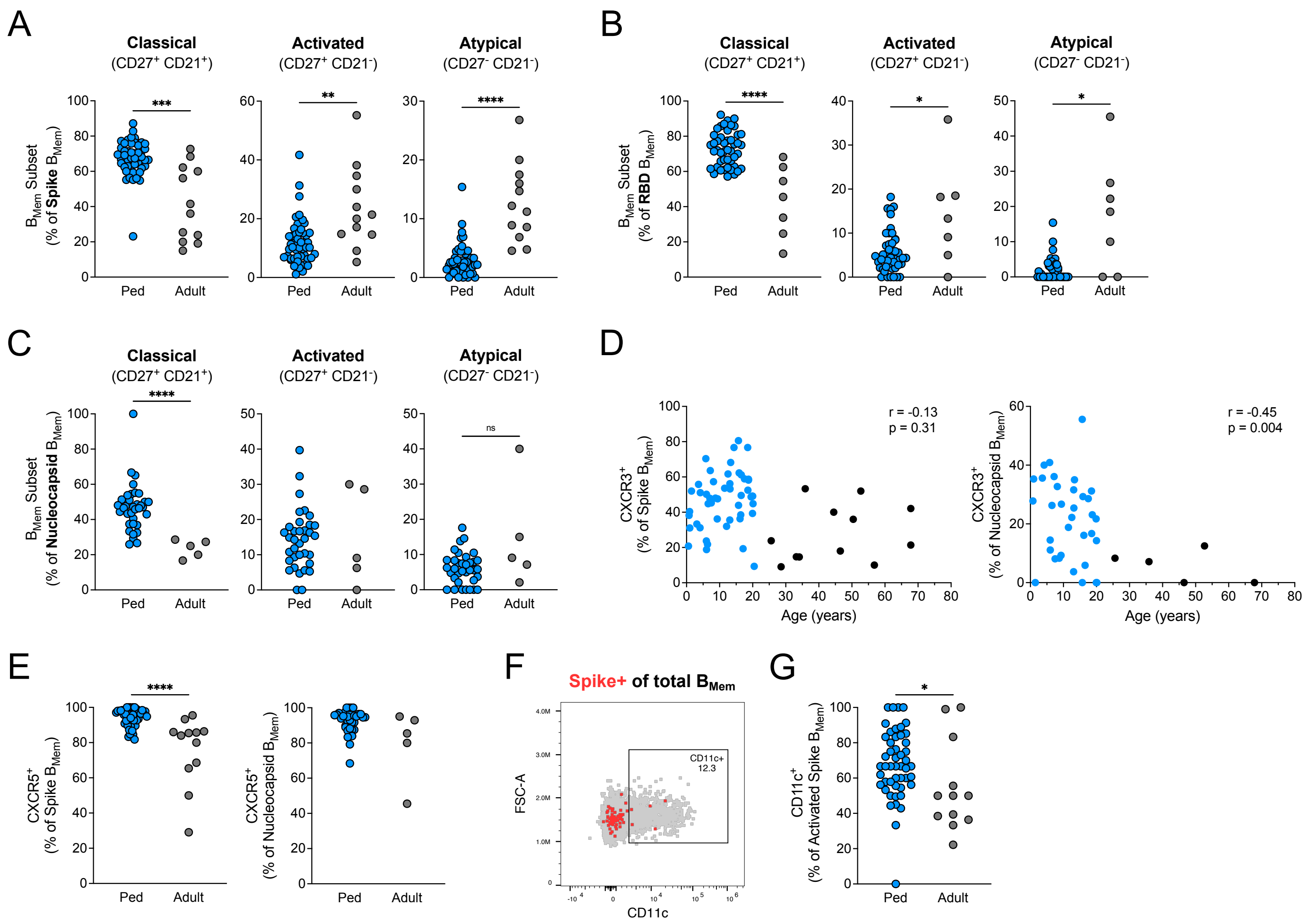

Supplemental Figure 7. SARS-CoV-2-specific memory B cell subsets in children and adults

(**A-C**) Frequencies of classical (left), activated (middle), and atypical (right) memory B cells indicated as percent of (**A**) Spike-binding memory B cells, (**B**) RBD-binding memory B cells, or (**C**) Nucleocapsid-binding memory B cells. (**D**) Correlations of CXCR3+ memory B cells as percent of Spike (left), and Nucleocapsid (right) binding memory B cells with age in the pediatric group. (**E**) Frequencies of CXCR5+ memory B cells indicated as percent of Spike or Nucleocapsid binding memory B cells. (**F**) Representative flow cytometry plot showing CD11c staining on SARS-CoV-2 specific memory B cells. (**G**) Frequency of CD11c+ activated memory B cells indicated as percent of Spike memory B cells. n=50 in the pediatric group; n=12 in the adult group. "Ped" in **B-E** represents the pediatric group. 'P' values for **A-C**, **E**, and **G** were calculated by Mann-Whitney test, and are indicated as *p<0.05, **p<0.01, ***p<0.001, ****p<0.0001; non-significant 'p' values are not shown. 'r' in **D** indicates Spearman correlation coefficient.

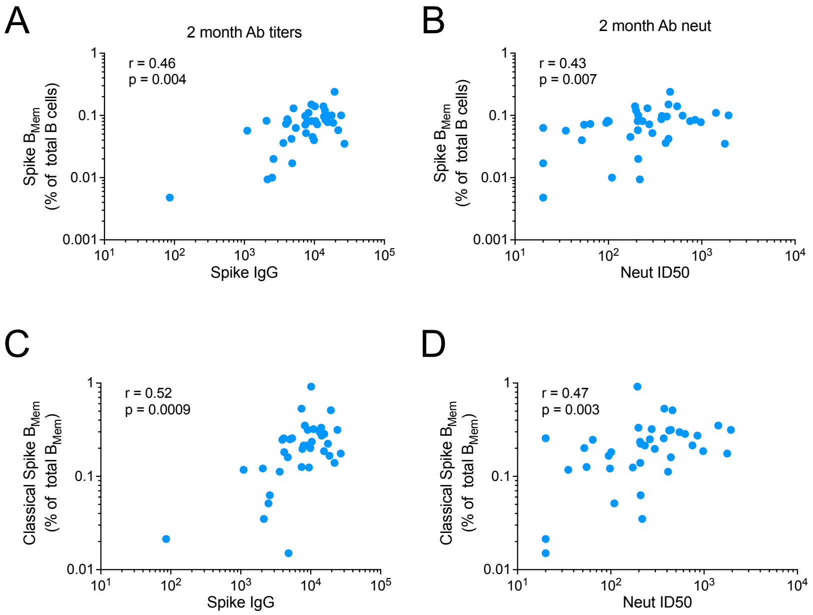

Supplemental Figure 8. Correlation of pediatric Spike memory B cell frequencies with humoral responses

(**A**) Correlation of Spike IgG titers as determined by BAMA assay at 2-months post-infection with spike memory B cells indicated as percent of total B cells. (**B**) Correlation of Neut ID50 titers at 2-months post-infection with spike memory B cells indicated as percent of total B cells. (**C**) Correlation of Spike IgG titers as determined by BAMA assay at 2-months post-infection with classical memory B cells indicated as percent of total memory B cells. (**D**) Correlation of Neut ID50 titers at 2-months post-infection with classical memory B cells indicated as percent of total memory B cells. 'r' indicates the Spearman correlation coefficient.

Supplemental Table 1: Study Cohort Description.

Characteristics N (%)	0-3 years	4-11 years	12-15 years	16-20 years	Pediatric (0-20 years)	Adults (≥21 years)
	27	42	30	33	132	51
**Age (years), median (IQR)**	2.9 (1.2, 3.6)	7.5 (6.1, 9.3)	13.8 (13.1, 14.9)	17.9 (16.7, 19.5)	11.1 (5.7, 16.0)	42.4 (34.0, 56.4)
**Sex**						
Female	13 (48%)	15 (36%)	17 (57%)	16 (48%)	61 (46%)	25 (49%)
Male	14 (52%)	27 (64%)	13 (43%)	17 (52%)	71 (54%)	26 (51%)
**Race/Ethnicity**						
Non-Hispanic White	5 (19%)	3 (7%)	6 (20%)	6 (8%)	20 (15%)	32 (63%)
Non-Hispanic Black	3 (11%)	6 (14%)	4 (13%)	1 (14%)	14 (11%)	9 (18%)
Hispanic or Latino	17 (63%)	33 (79%)	20 (67%)	26 (78%)	96 (73%)	1 (2%)
Other race/ethnicity	2 (7%)	0	0	0	2 (2%)	9 (18%)
**Comorbidities**	7 (26%)	25 (40%)	18 (40%)	19 (42%)	50 (38%)	17 (33%)
Asthma	1 (4%)	4 (10%)	1 (3%)	3 (9%)	9 (7%)	6 (12%)
Obesity	4 (15%)	14 (33%)	10 (33%)	10 (30%)	38 (29%)	4 (8%)
Other comorbidities	3 (11%)	2 (5%)	3 (10%)	4 (12%)	12 (9%)	12 (24%)
**SARS-CoV-2 infection status**						
Uninfected	4 (15%)	3 (7%)	3 (10%)	5 (15%)	15 (11%)	8 (16%)
Infected	23 (85%)	39 (93%)	27 (90%)	28 (85%)	117 (89%)	43 (84%)
Viral load, $\log_{10,}$ median (IQR)	5.1 (2.4, 5.6)	4.0 (3.0, 5.7)	4.3 (3.2, 5.7)	4.4 (3.1, 5.2)	4.4 (3.0, 5.6)	4.6 (3.7, 6.1)
Infected and symptomatic	21 (91%)	28 (72%)	22 (81%)	23 (82%)	94 (80%)	42 (98%)
Days of symptoms prior to sampling, median (IQR)	7 (5, 9)	8 (7, 9)	7 (5, 10)	10 (8, 11)	8 (6, 10)	8 (4, 20)
Fever	10 (48%)	12 (43%)	15 (68%)	15 (65%)	52 (55%)	27 (64%)
Cough	10 (48%)	15 (54%)	6 (27%)	17 (74%)	48 (51%)	31 (74%)
Shortness of breath	1 (5%)	1 (4%)	3 (14%)	4 (17%)	9 (10%)	15 (36%)
Sore throat	1 (5%)	5 (18%)	6 (27%)	9 (39%)	21 (22%)	17 (40%)
Rhinorrhea	8 (38%)	6 (21%)	2 (9%)	6 (26%)	22 (23%)	21 (50%)
Congestion	4 (19%)	7 (25%)	5 (23%)	4 (17%)	20 (21%)	23 (55%)
Other symptoms	4 (19%)	4 (14%)	6 (27%)	5 (22%)	19 (20%)	40 (95%)

**Comorbidity data were unavailable for 14 of the adult participants.*

Supplemental Table 2: Sub-Cohort Characteristics by Analysis Type.

Analysis Cohort Characteristics	T cell analyses (n=175)	B cell analyses (n=62)
0-3 years, n (%)	25 (14%)	7 (11%)
4-11 years, n (%)	40 (23%)	16 (26%)
12-15 years, n (%)	30 (17%)	11 (18%)
16-20 years, n (%)	31 (18%)	16 (26%)
21+ years, n (%)	49 (28%)	12 (19%)
Infected, n (%)	152 (87%)	62 (100%)
Symptomatic infection, n (%)	135 (86%)	52 (84%)

Supplemental Table 3: T Cell Antibody Panel.

Reagent	Clone	Source (Catalog #)	Dilution
CXCR5 AF700	J252D4	Biolegend (356915)	1:200
CXCR3 BV605	G025H7	Biolegend (353728)	1:200
CCR7 BV711	G043H7	Biolegend (353228)	1:100
CCR6 BUV496	11A9	BD Biosciences (612948)	1:200
ICOS BUV563	DX29	BD Biosciences (741421)	1:150
PD-1 BUV737	EH12.1	Biolegend (612791)	1:200
4-1BB BV421	4B4-1	Biolegend (309820)	1:200
CD69 FITC	FN50	Biolegend (310904)	1:250
CD40L PE-DAZZLE594	24-31	Biolegend (310840)	1:500
OX40 APC	Ber-ACT35	Biolegend (350008)	1:100
CD3 BUV395	UCHT1	BD Biosciences (563546)	1:200
CD38 BUV661	HIT2	BD Biosciences (612969)	1:100
CD8 BUV805	SK1	BD Biosciences (612889)	1:800
CD14 BV510	63D3	Biolegend (367124)	1:100
CD16 BV510	3G8	Biolegend (302048)	1:200
CD20 BV510	2H7	Biolegend (302340)	1:50
CD45RA BV570	HI100	Biolegend (304132)	1:400
CD27 BV785	O323	Biolegend (302832)	1:100
CD4 APC-FIRE810	SK3	Biolegend (344662)	1:50
TNFA EF450	Mab11	Thermo Fisher (48-7349-42)	1:50
IL-2 BV650	MQ1-17H12	Biolegend (500334)	1:50

Supplemental Table 4: Summary Statistics for Cell Counts Included in Spike-binding B_{Mem} Cell Analyses.

Pediatric Summary		Adult Summary	
Samples (N)	49	Samples (N)	12
Spike-specific Cell # (GeoMean)	125	Spike-specific Cell # (GeoMean)	61
Spike-specific Cell # (ArithMean)	170	Spike-specific Cell # (ArithMean)	109
Spike-specific Cell # (Median)	135	Spike-specific Cell # (Median)	43.5
Spike-specific Cell # (Range)	13-602	Spike-specific Cell # (Range)	20-525
Samples <20 spike B_{Mem}: n (%)	1 (2.04)	Samples <20 spike B_{Mem}: n (%)	0 (0)

Supplemental Table 5: Memory B Cell Antibody Panel.

Reagent	Clone	Source (Catalog #)	Dilution
IgG BUV395	G18-145	BD Biosciences (564229)	1:100
CXCR5 BUV496	RF8B2	BD Biosciences (741115)	1:200
CD19 BUV563	SJ25C1	BD Biosciences (612916)	1:200
CD79b BUV615	CB3-1	BD Biosciences (751380)	1:50
CD95 BUV737	DX2	BD Biosciences (612790)	1:200
CXCR3 BUV805	1C6	BD Biosciences (742048)	1:50
IgD Pacific Blue	IA6-2	Biolegend (348224)	1:50
CD20 BV510	2H7	Biolegend (302340)	1:100
IgM BV570	MHM-88	Biolegend (314517)	1:200
CD27 BB515	M-T271	BD Biosciences (564642)	1:200
IgA VioBright-FITC	IS11-8E10	Miltenyi Biotech (130-113-480)	1:400
CD3 PerCP	SK7	Biolegend (344814)	1:100
CD14 PerCP	63D3	Biolegend (367152)	1:200
CD16 PerCP	3G8	Biolegend (302030)	1:200
CD56 PerCP	HCD56	Biolegend (318342)	1:200
CD71 PE-Dazzle594	CY1G4	Biolegend (334120)	1:200
CD11c PE-Cy5	3.9	Biolegend (301610)	1:200
CD21 AF700	Bu32	Biolegend (354918)	1:50
CD38 APC-Fire810	HIT2	Biolegend (303550)	1:200
